# The Therapeutic Potential of Dihydroartemisinin in Cancer Treatment

**DOI:** 10.3390/ijms27083420

**Published:** 2026-04-10

**Authors:** Zhaochuan Hu, Shuai Zhang, Yongqi Shi, Yunlei Song, Dan Miao, Wenhe Xiong, Jiaying Guo, Yumao Jiang

**Affiliations:** Ministry of Education, Jiangxi Provincial Key Laboratory of Tissue Engineering, Key Laboratory of Prevention and Treatment of Cardiovascular and Cerebrovascular Diseases, Scientific Research Center, Gannan Medical University, Ganzhou 341000, China; huzhaochuan@gmu.edu.cn (Z.H.); zhangshuai@gmu.edu.cn (S.Z.); 14768068632@163.com (Y.S.); syl@gmu.edu.cn (Y.S.); miaodan2015@163.com (D.M.); xiongwenhe@gmu.edu.cn (W.X.); guojiaying@gmu.edu.cn (J.G.)

**Keywords:** dihydroartemisinin, cancer therapy, hybrid compounds, combination therapy, drug delivery systems

## Abstract

Dihydroartemisinin (DHA), the active metabolite of artemisinin derivatives, is a clinically established antimalarial agent that has recently gained significant attention for its anticancer properties. This review systematically examines the molecular mechanisms underlying DHA’s antitumor effects and explores innovative strategies to enhance its bioavailability and therapeutic efficacy. DHA demonstrates substantial potential in combination therapies with conventional clinical agents, with its broad anticancer applications being strongly supported by both preclinical and clinical evidence. Furthermore, this article outlines future research directions, discusses challenges in clinical translation, and summarizes current scientific approaches addressing these limitations. Collectively, this review highlights DHA’s promising role in cancer treatment and provides a foundation for developing improved therapeutic strategies.

## 1. Introduction

Cancer remains a major global public health challenge, causing nearly ten million deaths annually. According to projections from the International Agency for Research on Cancer, the global cancer burden is expected to rise to 28.4 million new cases per year by 2040, primarily due to population aging and lifestyle changes [1]. Significant challenges in cancer treatment persist, including tumor heterogeneity [2], drug resistance [3], metastasis [4], recurrence [4], and damage to healthy tissues [5]. While targeted therapies and immunotherapies have provided breakthrough treatments for some patients, limitations such as adverse effects, high costs, restricted indications, and acquired resistance remain pressing concerns. In this context, natural products offer promising alternatives for anticancer drug development due to their multitarget mechanisms and favorable toxicity profiles, presenting new opportunities for clinical cancer management.

**Figure 1:**
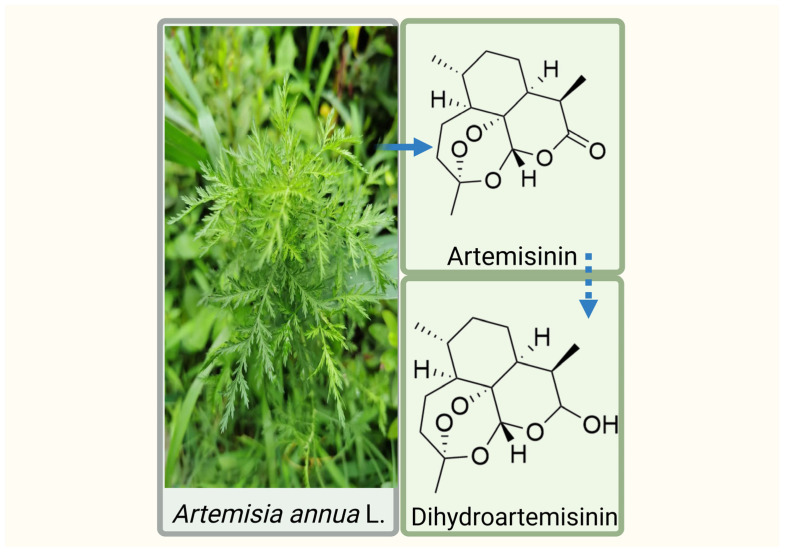
*Artemisia annua* L. and chemical structures of artemisinin and dihydroartemisinin. Created in BioRender. Yumao, J. (2026) https://BioRender.com/dsbs69b, accessed on 10 April 2026.

*Artemisia annua* L., commonly known as sweet wormwood, is an annual herbaceous plant belonging to the genus Artemisia within the Asteraceae family. Its primary bioactive constituent, artemisinin—a sesquiterpene lactone bearing a critical endoperoxide bridge—is metabolized in vivo to its more active derivative, dihydroartemisinin (DHA, C_15_H_24_O_5_) [6] (Figure 1). Beyond its well-established antimalarial efficacy [7], emerging pharmacological evidence has unveiled a wide spectrum of activities for DHA, including antitumor [8], anti-inflammatory [9], antiviral [10], and antifibrotic [11] effects. Most notably, its antitumor properties have garnered escalating attention over recent years. The unique endoperoxide moiety, which is essential for its parasiticidal action, also appears to mediate cancer cell cytotoxicity through mechanisms such as oxidative stress induction, distinguishing it from many conventional chemotherapeutics.

In this review, we critically examine the anticancer mechanisms and clinical potential of DHA, systematically exploring its multifaceted roles in inhibiting proliferation, inducing cell death, and suppressing metastasis. Furthermore, we investigate rational combination strategies with established therapies to provide new directions for anticancer drug development and inform the design of novel clinical treatment paradigms.

## 2. Mechanism of DHA’s Antitumor Effects

**Figure 2:**
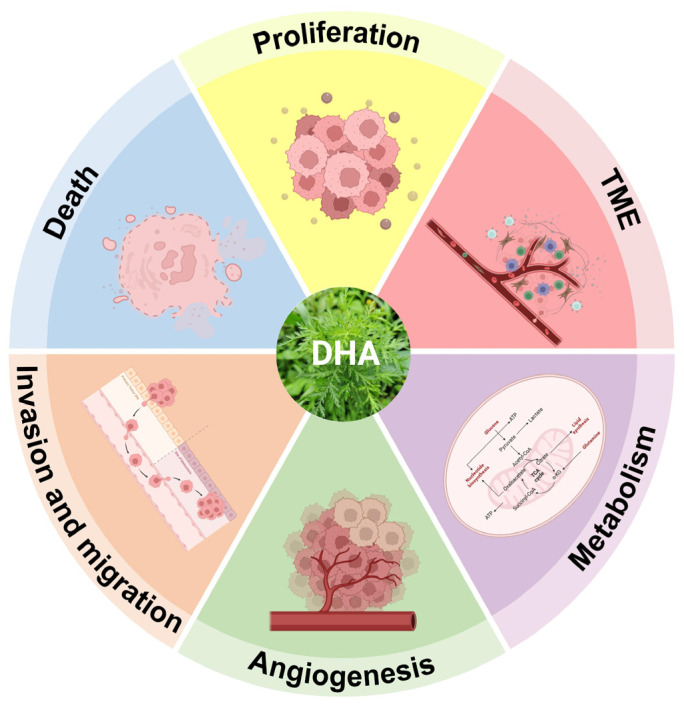
Pharmacological activity of dihydroartemisinin. Created in BioRender. Yumao, J. (2026) https://BioRender.com/7s7cmpc, accessed on 10 April 2026.

As a novel antitumor candidate, DHA exerts effects through multiple targets, stages, and pathways (Figure 2). This section reviews the molecular mechanisms underlying DHA’s antitumor activity to support its application in cancer therapy.

### 2.1. Inhibition of Tumor Cell Proliferation

Tumor cell proliferation is defined by uncontrolled, autonomous growth driven by genetic mutations that allow cells to evade normal regulatory signals. Key features include dysregulated cell cycle control, continuous cell division, and clonal expansion. DHA suppresses tumor cell proliferation by targeting these fundamental processes.

The cell cycle is a core regulatory mechanism that controls growth, development, proliferation, and maintenance of normal cellular function. Its orderly progression depends on coordinated activation of cyclins and cyclin-dependent kinases (CDKs).

**Figure 3:**
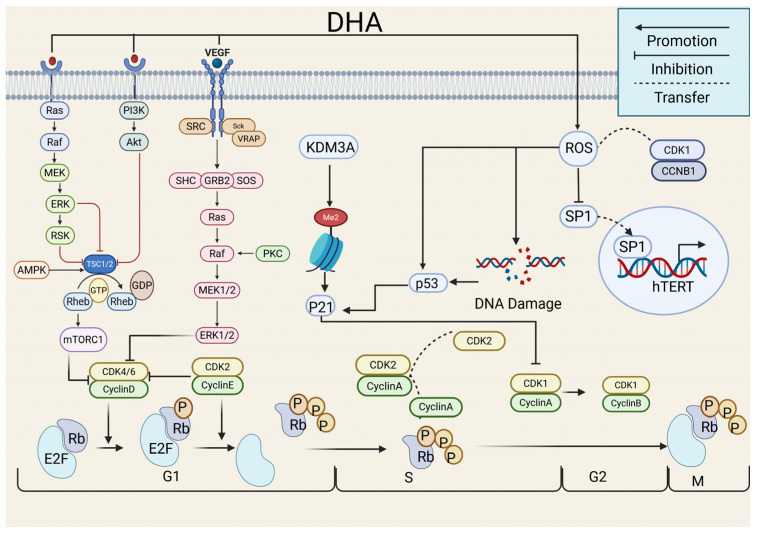
Anti-proliferation mechanism of dihydroartemisinin. Dihydroartemisinin regulates the CDK-cyclin complex by downregulating ERK1/2 and upregulating the AMPK, promoting p21 transcription, inducing DNA damage, and increasing ROS content to prevent cell cycle progression. Dihydroartemisinin suppresses *hTERT* transcription by activating the ROS/SP1 signaling axis, thereby impairing telomerase function. Created in BioRender. Yumao, J. (2026) https://BioRender.com/5q4f2hh, accessed on 10 April 2026.

DHA regulates the entire cell cycle through multiple pathways, serving a central role in inhibiting tumor proliferation (Figure 3). Studies indicate that in non-small cell lung cancer (NSCLC), DHA suppresses signal transducer and activator of transcription 3 (STAT3) phosphorylation through downregulation of ROR1 expression, leading to reduced CDK2/4 and Cyclin D1/E1 levels and impaired G0/G1 phase transition [12]. In rhabdomyosarcoma cells, DHA activates AMP-dependent protein kinase (AMPK) to inhibit the mTORC1 signaling pathway, downregulate Cyclin D expression, and induce G1 phase arrest [13]. When combined with N-alkyl-triphenylvinylpyridine, DHA also induces G1 phase arrest by suppressing Cyclin D expression through inhibition of the ERK1/2 pathway [14]. DHA downregulates histone demethylase KDM3A [14] or activates the p53 gene [15], enhancing p21 transcription and promoting p21 binding to the Cyclin D/CDK complex. DHA can markedly reduce CDK4 and Cyclin D1 expression [16], ultimately resulting in G1/S phase arrest in lung and gastric cancer cells. Beyond G1/S regulation, DHA induces G2/M phase arrest in esophageal carcinoma cells [17] and hepatocellular carcinoma (HCC) cells [18] by inhibiting CDK1/cyclin B1 (CCNB1) activity. Similarly, DHA triggers G2/M arrest in melanoma cells by downregulating β-tubulin 4A [19].

Telomeres are specialized nucleoprotein complexes located at chromosome ends. They protect chromosomal structure, prevent DNA loss, and maintain genomic stability. Telomeres shorten with each cell division and trigger cellular senescence or apoptosis once critically shortened. Telomerase is the main enzyme responsible for telomere elongation, and it is inactive in most somatic cells but abnormally activated in approximately 85% of malignant tumors [20]. DHA inhibits tumor growth by targeting telomerase. In esophageal carcinoma Eca-109 cells, DHA suppresses human telomerase reverse transcriptase (hTERT) transcription by activating the reactive oxygen species (ROS)/SP1 pathway, thereby reducing telomerase activity and shortening telomeres [21]. In addition, DHA-induced autophagy promotes lysosomal degradation of the telomere-binding protein TRF2, resulting in telomere dysfunction and activation of the DNA damage response [17]. Thus, DHA can interfere with the cell cycle process through multiple pathways to exert its anti-proliferative effects on tumors.

### 2.2. Induction of Tumor Cell Death

**Figure 4:**
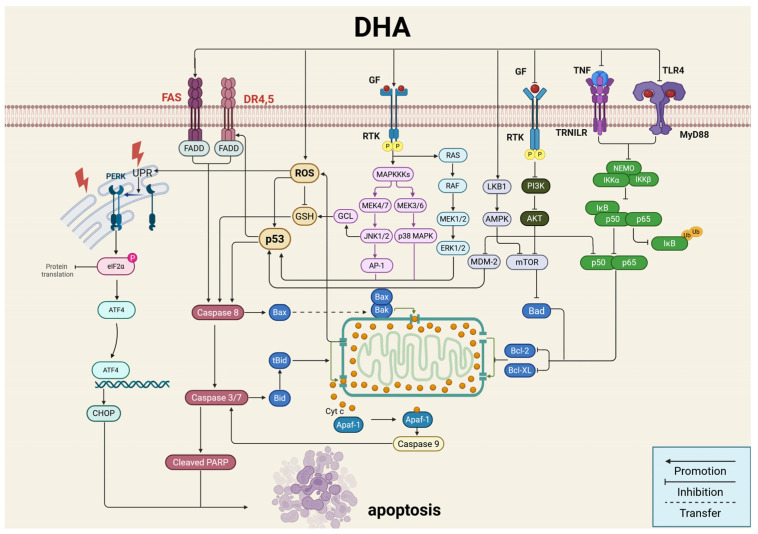
Dihydroartemisinin induces tumor apoptosis by activating mitochondrial and endoplasmic reticulum stress-mediated apoptotic pathways. Created in BioRender. Yumao, J. (2026) https://BioRender.com/sku7zqv, accessed on 10 April 2026.

**Figure 5:**
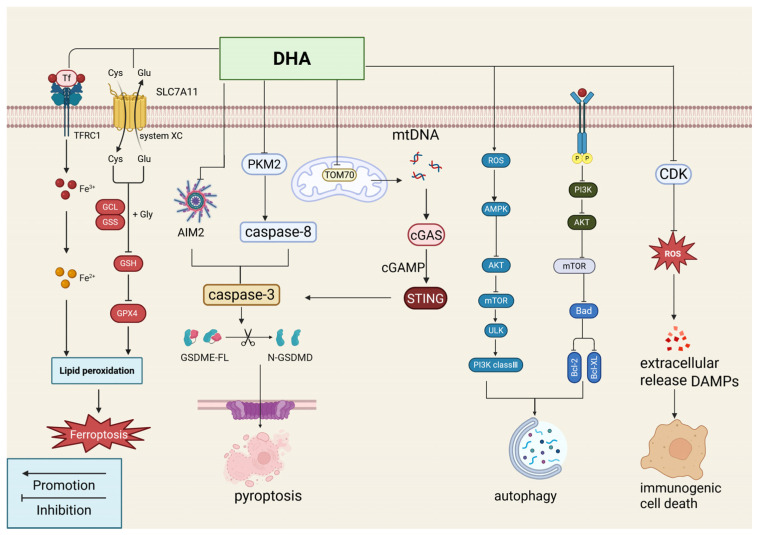
The mechanism of dihydroartemisinin-induced ferroptosis, autophagic death, immunogenic death, and pyroptosis. Dihydroartemisinin induces ferroptosis by increasing ROS content and down-regulating GPX4 expression. It can also induce autophagic cell death via the AMPK/mTOR pathway, promote immunogenic cell death through the pathway of reduced CDK expression leading to ROS accumulation and the release of DAMPs, and induce pyroptosis via inhibition of PKM2 and activation of the Caspase-8/3 pathway, thereby upregulating GSDME. Created in BioRender. Yumao, J. (2026) https://BioRender.com/hfcwc8x, accessed on 10 April 2026.

Programmed cell death (PCD) is a genetically regulated form of cell elimination mediated by defined molecular pathways. Together with immunogenic cell death (ICD), PCD forms a major mechanism involved in cancer treatment. Growing evidence demonstrates that DHA induces tumor cell death by regulating these pathways (Figure 4 and Figure 5). This section summarizes key molecular mechanisms by which DHA regulates PCD and ICD, as well as its related antitumor potential.

#### 2.2.1. Apoptosis

The mitochondrial pathway is the primary mechanism of intrinsic apoptosis. This pathway includes mitochondrial outer membrane permeabilization, release of cytochrome c into the cytoplasm, formation of apoptosomes with Apaf-1 and ATP/dATP, and initiation of the caspase cascade culminating in apoptosis. DHA shows strong antitumor potential by regulating this pathway. Studies demonstrate that DHA promotes apoptosis in prostate cancer (PCa) by modulating the Beclin-1/Bcl-2 interaction through the ROS/AMPK/mTOR pathway [22]. In ovarian cancer, DHA increases RECK expression, reduces Bcl-2 levels, and increases Bax and Cleaved Caspase-3 [23]. DHA also inhibits glioma growth by suppressing ERRα-mediated mitochondrial biogenesis, resulting in abnormal mitochondrial membrane potential in U87 cells [24]. Similar effects have been observed in oral squamous cell carcinoma (OSCC) [25] and melanoma [26]. Furthermore, in non-small cell carcinoma, the dihydroartemisinin-cinnamic acid hybrid compound 16g (compound **30**, Section 3.1) induces apoptosis in A549 cells by inhibiting the Akt/Bad pathway, causing mitochondrial depolarization and ROS accumulation [27]. DHA hybrids (compound **28**, Section 3.1) induce apoptosis in multiple tumor cell lines via the MAPK/JNK pathway and mitochondrial membrane depolarization [15], a mechanism similar to the novel DHA hybrid Mito-DHA (compound **40**, Section 3.1), which triggers bladder cancer cell apoptosis [28]. Notably, studies show that under normoxic conditions, DHA-mediated cytotoxicity in HCT116 colorectal cells depends on Bax and Bak expression, while under hypoxia, reduced ATP levels lower glutathione (GSH) concentrations and weaken antioxidant function, enhancing DHA-induced oxidative damage independently of Bax and Bak [29]. This suggests that DHA may help overcome tumor hypoxia and improve treatment outcomes.

The ER is responsible for protein folding. Disruption of ER function triggers ER stress and activation of the unfolded protein response to restore homeostasis. When damage persists, ER stress shifts toward apoptosis. DHA induces ER stress–mediated apoptosis to eliminate tumor cells. Studies show that in HCC, DHA promotes apoptosis by inhibiting COX-2-mediated resistance during ER stress while upregulating Bax and Caspase-3 [30]. In colon cancer cells, DHA dimers (compound **29**, Section 3.1) activate the PERK/eIF2α/CHOP pathway through a heme-dependent process to induce apoptosis independent of ROS [31]. Evidently, DHA can effectively intervene in the critical decision between “survival adaptation” and “programmed cell death” mediated by ER stress in tumor cells.

DHA also accelerates apoptosis in laryngeal carcinoma cells by downregulating osteopontin expression, suppressing YAP signaling, and reducing IL-6 expression [32].

In summary, intrinsic apoptosis is a key pathway through which DHA induces tumor cell death.

#### 2.2.2. Ferroptosis

Ferroptosis is an iron-dependent form of regulated cell death characterized by intracellular iron accumulation and the buildup of lipid peroxidation products, ultimately leading to plasma membrane damage and cell death. During ferroptosis, elevated iron levels promote reactive oxygen species generation through the Fenton reaction, resulting in the accumulation of lipid peroxides (LPO) that oxidize unsaturated fatty acids within cell membranes. DHA effectively induces ferroptosis in various tumor cells by disrupting iron homeostasis, promoting lipid peroxidation, and inhibiting cellular antioxidant systems. Studies report that in cervical cancer, DHA not only promotes lipid peroxidation and disrupts iron metabolism by increasing ROS, malondialdehyde, and LPO levels but also activates NCOA4-mediated ferritinophagy to expand the labile iron pool (LIP), thereby intensifying Fenton reaction activity and initiating lipid peroxidation cascades [33]. Simultaneously, DHA suppresses the antioxidant system by depleting GSH, downregulating glutathione peroxidase 4 (GPX4) expression and activity, and impairing LPO clearance [33]. A similar GSH-depleting effect is observed in malignant peripheral nerve sheath tumors [34], although whether it can trigger ferroptosis remains to be verified. Additional studies indicate that in lung cancer cells, DHA reduces cysteine uptake and GSH synthesis by inhibiting the PRIM2/SLC7A11 axis, thereby exacerbating oxidative stress and inducing ferroptosis [35]. In liver cancer, DHA induces ferroptosis by upregulating ChaC glutathione-specific gamma-glutamylcyclotransferase 1, which further reduces GSH synthesis and downregulates GPX4 expression [36]. A DHA hybrid bearing a 4-Cl-phenylcarbamate group (compound **28**, Section 3.1) also downregulates STAT3 to suppress GPX4 expression, increasing ROS accumulation and GSH depletion to trigger ferroptosis in colon cancer cells [15]. It is evident that ferroptosis represents a key pathway in the antitumor activity of DHA. However, distinct genetic backgrounds and metabolic characteristics of different cancers, and even different cell lines, determine their sensitivity or resistance to ferroptosis. Therefore, a tailored approach remains essential. At the same time, varying experimental conditions—including treatment protocols and model systems—must be considered when interpreting related conclusions.

On the other hand, ferroptosis-induced treatment resistance cannot be overlooked. Studies show that when DHA induces ferroptosis in glioblastoma cells, it increases the expression of heat shock protein family A (Hsp70) member 5, activating a compensatory ferroptosis feedback pathway. This response elevates GPX4 expression and reverses DHA-induced lipid peroxidation. As a result, glioblastoma cells are protected from ferroptosis, thereby reducing the anticancer efficacy of DHA [37]. Moreover, ROS and lipid peroxidation products associated with ferroptosis, such as 4-HNE, may impair dendritic cell maturation, exerting an inhibitory effect on antitumor immunotherapy and limiting complete tumor clearance [38]. Additionally, ferroptosis resistance may also contribute to tumor metastasis. Research has revealed that in gastric cancer patients with peritoneal metastasis, galectin-1 expression is significantly elevated. It enhances the ferroptosis defense capacity of gastric cancer cells by activating the PI3K/Akt/Nrf2/Heme Oxygenase-1 (HO-1) signaling pathway, thereby promoting metastasis formation. DHA can inhibit galectin-1 expression and reverse this resistance mechanism, suggesting its potential in overcoming ferroptosis resistance [39].

It is clear that ferroptosis exhibits a double-edged sword effect in cancer therapy: while it can eliminate tumor cells, it may also trigger treatment resistance. Future research should focus on precisely modulating ferroptosis and blocking its adaptive feedback mechanisms, fully leveraging DHA’s dual potential as both a ferroptosis inducer and a resistance reversal agent, while exploring combined intervention strategies to achieve superior antitumor efficacy.

#### 2.2.3. Autophagic Cell Death

Autophagy is a highly conserved catabolic process that typically serves as a protective mechanism for tumor cells in response to internal and external stress. However, excessive activation can trigger autophagic cell death. DHA induces autophagy-dependent cell death in multiple tumor types. Studies indicate that in HCC cells, DHA upregulates the autophagy-related protein Beclin-1 by downregulating histone methyltransferase-associated zinc finger protein, thereby enhancing transcription of the E3 ubiquitin ligase TRIM50 and inducing autophagy-mediated cell death [40]. In PCa, DHA induces ROS bursts, activates AMPK, inhibits mTORC1, and weakens the binding between Beclin-1 and Bcl-2 while enhancing its interaction with Vps34, thereby initiating autophagy and leading to excessive degradation of organelles, such as mitochondria [22]. Similarly, in cervical cancer, DHA increases Ser70 phosphorylation of Bcl-2, reducing its binding to Beclin-1; at the same time, it inhibits Ser2448 phosphorylation of mTOR, thereby increasing Beclin-1 expression and activating autophagy [41]. However, autophagy also represents a double-edged sword in cancer therapy, as this normally protective process frequently contributes to drug resistance and requires continued investigation.

#### 2.2.4. Pyroptosis

Pyroptosis is a programmed cell death pathway mediated by inflammatory caspase cleavage and activation of the Gasdermin family proteins, resulting in plasma membrane perforation, cellular swelling, membrane rupture, and the release of proinflammatory cytokines. DHA effectively induces pyroptosis in multiple cancer types. Studies show that in lung cancer, DHA downregulates the mitochondrial outer membrane transporter TOM70, causing mitochondrial DNA damage and subsequent mtDNA release into the cytosol, where it activates the cGAS-STING-NLRP3 pathway to initiate pyroptosis [8]. In breast cancer, DHA upregulates the AIM2 inflammasome and activates Caspase-3 to cleave Gasdermin E (GSDME) [42]. Together, these mechanisms demonstrate the multitargeted anticancer effects of DHA. Therefore, elucidating DHA-related target molecules is of major clinical significance for developing treatment strategies that trigger pyroptosis in tumor cells.

#### 2.2.5. Immunogenic Cell Death

ICD is a regulated form of cell death characterized by the release of damage-associated molecular patterns (DAMPs), which activate the adaptive immune system and promote antitumor immunity. DHA induces ICD in tumor cells through multiple pathways. Studies show that DHA reduces CDK expression, induces sustained ROS accumulation, and promotes the release of DAMPs, including CRT and Hsp70, to trigger ICD in liver cancer cells [43]. In lung cancer cells, DHA induces ER stress and activates the unfolded protein response along with DNA damage, resulting in surface exposure of immunogenic markers such as calreticulin [44]. Similarly, the dihydroartemisinin hybrid T-D (compound **41**, Section 3.1), specifically localizes to mitochondria, generating strong ROS production that disrupts mitochondrial membrane potential and induces ER stress, ultimately triggering ICD in breast cancer cells [45]. Because ICD can remodel the immunosuppressive tumor microenvironment, overcome immune tolerance, and reduce the risk of recurrence and metastasis, DHA and its derivatives may have broader therapeutic potential beyond direct tumor cell killing. By inducing ICD, they activate the host immune system to eliminate cancer cells, providing a promising avenue for developing novel combination immunotherapy strategies.

### 2.3. Inhibition of Tumor Cell Invasion and Migration

**Figure 6:**
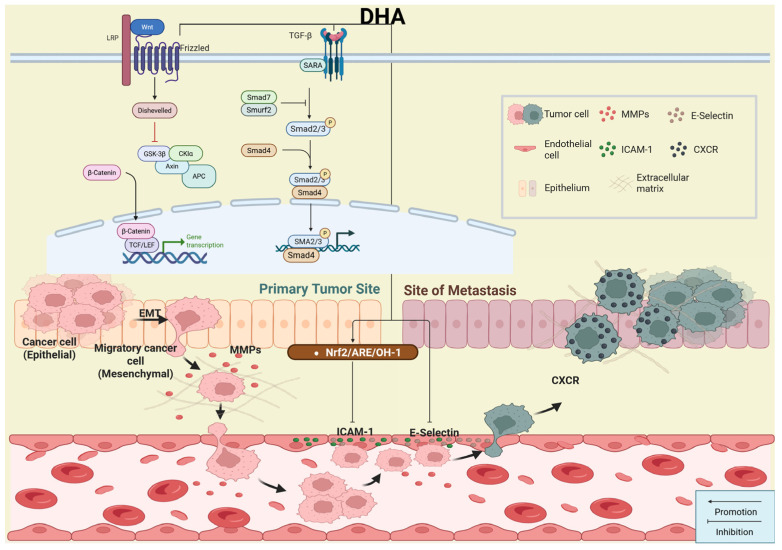
Mechanism of dihydroartemisinin against invasion and migration. Dihydroartemisinin reverses EMT by regulating Wnt/β-catenin and TGF-β1/Smad signaling, thereby reducing migratory ability. Created in BioRender. Yumao, J. (2026) https://BioRender.com/5w848m7, accessed on 10 April 2026.

Tumor invasion and metastasis are fundamental malignant features of cancer. They involve a complex multi-step process in which cancer cells detach from the primary tumor, degrade the basement membrane and extracellular matrix, invade adjacent tissues, enter the blood or lymphatic system, and disseminate to distant sites to establish secondary tumors. This cascade includes loss of cell–cell adhesion, increased migratory ability, secretion of proteolytic enzymes, intravasation, and survival during circulation. Metastasis remains the primary cause of treatment failure and mortality in cancer. Therefore, targeting invasion and migration pathways is a critical goal in cancer therapy. DHA inhibits tumor metastasis through multiple molecular mechanisms (Figure 6).

#### 2.3.1. Inhibition of Epithelial–Mesenchymal Transition

Epithelial–mesenchymal transition (EMT) is a biological process in which epithelial cells acquire mesenchymal properties, gain mobility, and develop metastatic potential. DHA suppresses tumor metastasis by inhibiting EMT. Studies report that in PCa, DHA upregulates the transcription factor NR2F2, suppresses N-cadherin and vimentin expression, and promotes E-cadherin expression [46]. In esophageal carcinoma Eca109 cells, DHA increases DAB2IP expression through NFIC-dependent mechanisms [47] and simultaneously inhibits the Akt/mTOR pathway [48], collectively upregulating E-cadherin and downregulating vimentin. In NSCLC, DHA increases miR-497-5p expression, reduces SOX5 levels, upregulates E-cadherin, and downregulates N-cadherin [49]. In gastric cancer, DHA downregulates terminal anchoring polymerase to inhibit the Wnt/β-catenin signaling pathway, thereby suppressing EMT and cell migration [50]. In medullary thyroid carcinoma, DHA reduces IL-6 expression, promotes YAP phosphorylation, and inhibits YAP/TAZ protein expression by activating the Hippo pathway, thereby increasing E-cadherin and suppressing EMT [51]. In glioblastoma, DHA upregulates ecDNA-BASP1, downregulates N-cadherin and vimentin, and increases E-cadherin expression [52]. Additionally, TGF-β binding to its receptor induces Smad2/3 phosphorylation and nuclear translocation, thereby increasing the expression of CDKN1A-binding zinc finger protein 1 (CIZ1). This elevates Snail expression and decreases E-cadherin levels, promoting tumor metastasis. Intervention in the TGF-β1/Smad pathway may therefore represent a key anti-metastatic strategy. Consistent with this hypothesis, studies in breast cancer show that DHA inhibits the TGF-β1/Smad pathway and its downstream effector CIZ1, thereby influencing extracellular matrix remodeling [53]. However, given TGF-β’s ubiquitous presence across tissues, modulation of this pathway may cause substantial adverse effects. Thus, future research should focus on tumor-specific mechanisms of DHA action.

#### 2.3.2. Downregulation of Matrix Metalloproteinase Activity

Compared to normal cells, tumor cells show increased secretion of matrix metalloproteinases, which degrade the extracellular matrix and basement membrane to support invasion and migration. Studies demonstrate that in breast cancer, DHA inhibits MMP-2 and MMP-9 expression by suppressing the PI3K/AKT pathway and preventing HIF-1α activation, while reduced NF-κB phosphorylation further contributes to the inhibition of MMP-mediated signaling [54]. In gastric cancer, DHA downregulates MMP14 expression and limits degradation of extracellular matrix components, thereby suppressing tumor migration and invasion [55]. These findings indicate that DHA inhibits metastasis by suppressing MMPs through multiple pathways.

Beyond these mechanisms, DHA also suppresses IL-6 and hypoxia-induced laryngeal cancer metastasis within the tumor microenvironment by inhibiting STAT3 activation in tumor stem cells, downregulating MMP-9, and increasing E-cadherin expression [56]. In addition, DHA reduces Ras-associated GTP-binding protein B expression both in vivo and in vitro, suppressing actin cytoskeletal remodeling and limiting the migration of Cal-27 cells in squamous cell carcinoma of the tongue [57]. Thus, DHA can inhibit tumor metastasis by suppressing MMP through multiple pathways.

### 2.4. Inhibition of Angiogenesis

**Figure 7:**
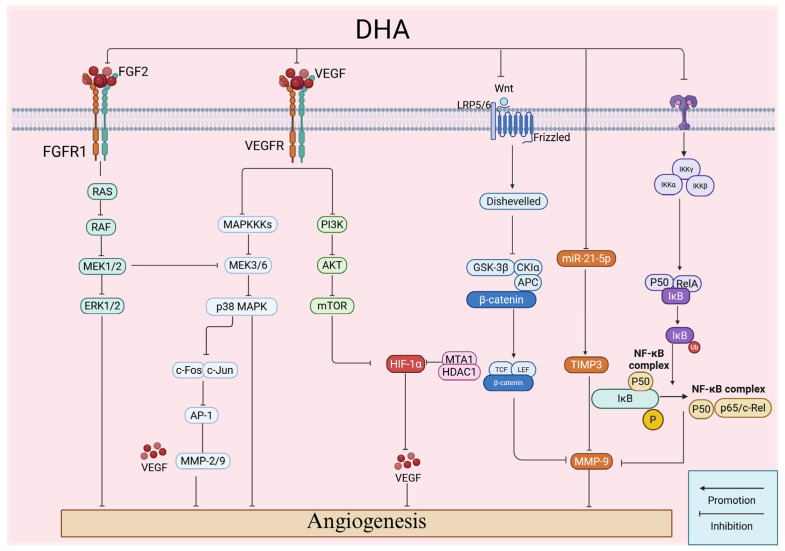
Mechanism of dihydroartemisinin against angiogenesis. Dihydroartemisinin inhibits angiogenesis by regulating RAS-RAF-MEK-ERK, PI3K/AKT/mTOR, GSK-3β, and NF-κB to downregulate the expression and interaction of VEGF, MMP-9, and VEGFR. Created in BioRender. Yumao, J. (2026) https://BioRender.com/w747y9k, accessed on 10 April 2026.

Tumor growth and distant metastasis require neovascularization to provide oxygen and nutrients. DHA inhibits angiogenesis in several cancer types through multiple pathways (Figure 7). Hypoxia within tumors activates HIF-1α, which triggers expression of pro-angiogenic genes and promotes VEGF secretion to support endothelial cell proliferation and migration. Studies report that in breast cancer, DHA inhibits the PI3K/AKT pathway, blocks HIF-1α activation and NF-κB phosphorylation, and reduces VEGF expression [54]. Similar results have been confirmed in melanoma [58]. Further research shows that in HCC, DHA targets ANXA2, thereby indirectly suppressing PI3K/AKT signaling and reducing VEGF secretion [59]. Beyond conventional angiogenesis, tumors can also form endothelial-independent vasculogenic mimicry (VM) structures to maintain blood supply. Reports show that in ovarian cancer, DHA exhibits dual inhibitory effects on angiogenesis and VM: on one hand, it blocks endothelial angiogenesis by suppressing VEGF secretion in ovarian cancer cells and VEGFR2 expression in endothelial cells; on the other hand, it reduces VEGF-A-induced VM formation while simultaneously interfering with both blood supply pathways [60]. In gastric cancer, DHA downregulates FGF2 expression and inhibits FGFR1-mediated activation of the Ras/MAPK and PI3K/AKT pathways [61]. This reduces activity of VM-associated proteins, including MMP2 and VE-cadherin, effectively blocking VM formation. In glioma models, DHA suppresses EphA2, leading to downregulation of MMP-2/3/9 and inhibition of VM networks [62]. IL-8 is a classical angiogenesis factor that promotes endothelial cell proliferation, migration, and tube formation. Studies indicate that DHA directly binds to JAK3, inhibiting phosphorylation at Y981, downregulating STAT5A, and suppressing IL-8 transcription, thereby inhibiting angiogenesis in esophageal squamous cell carcinoma [63]. Thus, DHA can suppress tumor angiogenesis through multiple pathways.

### 2.5. Regulation of Energy Metabolism

**Figure 8:**
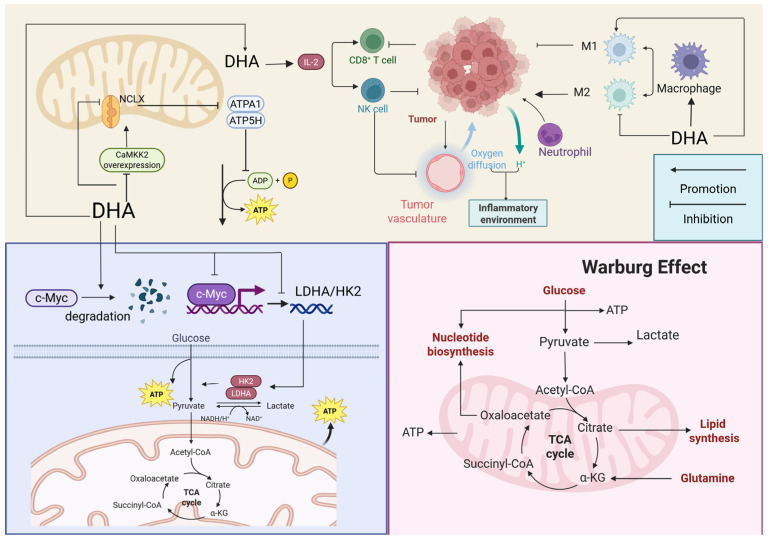
The mechanism of dihydroartemisinin in modulating the tumor microenvironment and reprogramming energy metabolism. Dihydroartemisinin disrupts the tumor microenvironment by inhibiting macrophage M2 polarization and reshaping the cytokine network. It also reprograms the glucose metabolic pathway by downregulating c-Myc transcription factor expression and suppressing CaMKK2 overexpression. Created in BioRender. Yumao, J. (2026) https://BioRender.com/19kjjmi, accessed on 10 April 2026.

Unlike normal cells, tumor cells adopt distinct metabolic strategies to support continuous proliferation. Even in the presence of oxygen, they accelerate glucose uptake and lactate production through aerobic glycolysis (the Warburg effect) to generate ATP and supply biosynthetic precursors. DHA suppresses tumor growth by targeting metabolic enzyme networks (Figure 8). Studies in NSCLC demonstrate that DHA significantly downregulates c-Myc and promotes its degradation, blocking its transcriptional activation of glycolytic enzyme genes, including LDHA and HK2 [64]. This inhibition interferes with rapid ATP production, which is essential for tumor growth. The liver, as the primary organ of glycogen metabolism, is central to metabolic reprogramming in HCC. DHA inhibits GLUT3 [65] and GLUT1 [66] expression by suppressing YAP1, thereby restraining the Warburg effect and reducing glucose uptake and lactate production. Additionally, DHA significantly impairs glucose uptake and disrupts glycolysis, thereby interfering with the energy metabolism of colorectal cancer (CRC) stem cells [67]. DHA also suppresses CaMKK2 overexpression, downregulates NCLX, and reduces activity of ATP synthase subunits ATP1A1 and ATP5H, ultimately decreasing ATP synthesis [68]. Similarly, DHA downregulates calnexin, reducing ATP synthase subunit levels, including ATP6V0B and ATP6V1F, thereby further inhibiting cellular energy metabolism and energy transfer [69]. These findings show that DHA modulates tumor energy metabolism through several regulatory pathways. However, the generalizability of this strategy across cancer types remains unclear, and underlying mechanisms require further investigation.

### 2.6. Modulating the Tumor Microenvironment

The tumor microenvironment is a complex and dynamic ecosystem surrounding tumor cells, comprising both cellular and non-cellular components. It provides structural support and serves as a regulatory hub for malignant progression. CAFs are stromal cells within the tumor microenvironment that promote tumor proliferation by secreting lactic acid. Reports show that DHA suppresses PDGF-BB secretion in OSCC, inhibiting fibroblast transformation into CAFs [70]. DHA also downregulates Serpin Family B Member 5 in pancreatic cancer, thereby affecting CAF-mediated regulation of the tumor microenvironment and suppressing tumor proliferation [71].

The tumor immune microenvironment constitutes a dynamic region within tumor tissue shaped by infiltrating immune cells, cytokine networks, and immunosuppressive molecules. Its defining feature is the formation of an immunosuppressive ecosystem that facilitates tumor immune escape and confers treatment resistance, making it a key target for modern cancer immunotherapy. DHA remodels this microenvironment through multiple mechanisms (Figure 8). Studies show that in NSCLC, DHA significantly promotes CD8^+^ T cell infiltration by downregulating B7-H3 [72] and enhances CD8^+^ T cell cytotoxicity while counteracting IL-10-mediated immunosuppression driven by regulatory T cells [26]. This regulatory mechanism exhibits pan-cancer universality. In pancreatic cancer (PANC-1, AsPC-1), PCa (PC-3), and CRC (HCT116) cells, DHA similarly significantly downregulates B7-H3 expression while universally upregulating MHC-I molecule levels, thereby synergistically enhancing tumor immunogenicity and T cell recognition capacity [73]. In triple-negative breast cancer (TNBC), DHA reduces PD-L1 protein levels by activating the IRE1/IKK1 signaling axis, inducing FoxO3a phosphorylation and ubiquitin-mediated degradation, thereby enhancing tumor cells’ sensitivity to T cell-mediated killing [74]. In HCC, DHA inhibits YAP1, reducing IL-18 expression [75] or suppresses histone lactylation [76], thereby alleviating immunosuppression. DHA-mediated inhibition of IL-8 secretion has also been observed in LPS-treated HT-29 cells [77]. Additionally, DHA decreases the recruitment and accumulation of tumor-associated macrophages by blocking CCL2–CCR2 binding. At the same time, it suppresses polarization toward the M2 phenotype while promoting M1 polarization, thereby lifting immunosuppression and counteracting lung cancer metastasis [78]. DHA also significantly inhibits invasion, migration, and angiogenesis in head and neck squamous cell carcinoma by blocking M2 macrophage polarization through inhibition of STAT3 phosphorylation [79]. Additionally, DHA can induce tumor-associated neutrophil dNB4 cells to polarize toward an anti-tumor N1 phenotype (upregulating TNF, IL-1β, PD-L1, NOX2, etc., while downregulating CEACAM8 and CLEC10A), thereby enhancing the immune response against HCC [80]. Thus, DHA exhibits substantial potential in tumor immunotherapy by enhancing antitumor immune activity, preventing immune evasion, and disrupting tumor-promoting inflammatory environments.

## 3. Enhancing the Potential of DHA in Cancer Treatment

Current tumor treatment options include surgery, radiotherapy, chemotherapy, photodynamic therapy, and immunotherapy. However, single-modality therapy has limited effectiveness due to high tumor heterogeneity. Tumor evolutionary adaptability readily induces drug resistance, and existing treatments often produce substantial toxic side effects. As the primary active metabolite of artemisinin derivatives in vivo, DHA shows strong antitumor activity alongside a favorable safety profile. Combination with current therapies offers a promising strategy to address these challenges. However, due to its peroxy bridge and lactone ring, DHA possesses low polarity and poor water solubility. Combined with its short half-life in vivo, these properties contribute to clinical limitations, including low bioavailability, uncontrolled release, and suboptimal pharmacokinetics. Therefore, molecular hybridization of its pharmacophore or development of novel delivery platforms has become an effective strategy to enhance antitumor efficacy and therapeutic value.

### 3.1. Hybridization of DHA

**Figure 9:**
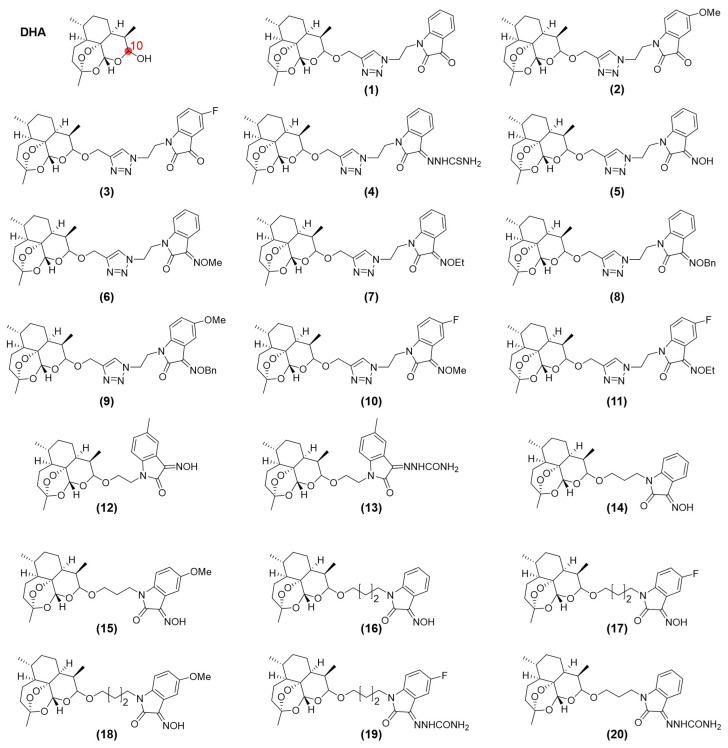
Structures of dihydroartemisinin and its hybrids (compounds **1**–**20**).

**Figure 10:**
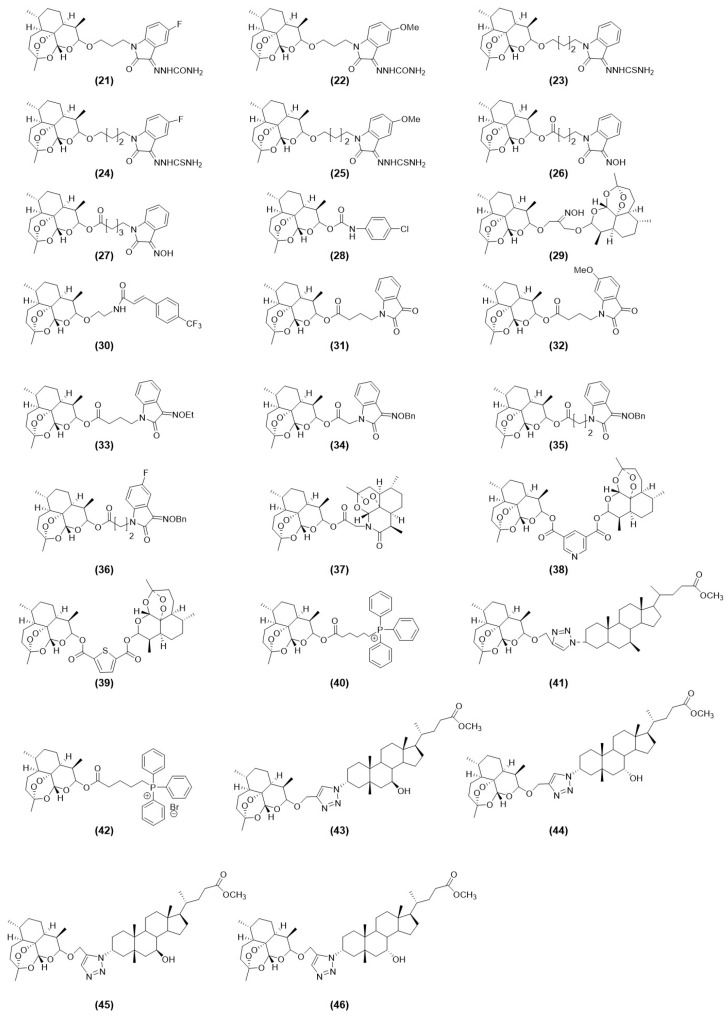
Structures of dihydroartemisinin and its hybrids (compounds **21**–**46**).

DHA has attracted considerable attention for its strong antitumor activity. Molecular hybridization couples the DHA scaffold with other bioactive pharmacophores, preserving the advantages of the parent molecule while improving efficacy, reducing toxicity, and overcoming drug resistance. Common pharmacophores include porphyrin, isophorone, and cinnamoyl derivatives, among others (Figure 9 and Figure 10) (Table S1). Notably, in the DHA structure, only the hydroxyl group is a relatively reactive modification site, while other sites exhibit low reactivity without disrupting the original structure. Therefore, in traditional methods, modifications are exclusively targeted at this site, typically by forming an ether bond with the hydroxyl group at the C10 position of DHA under the catalysis of BF_3_·OEt_2_, thereby introducing the hybrid group.

Hybrid DHA structures that incorporate diverse pharmacophores demonstrate superior biological activity through multiple mechanisms. Compared with unmodified DHA, these hybrids show stronger cytotoxicity across tumor types [15,27,28,31,81,82,83,84,85,86,87,88,89,90], enhanced anti-proliferative activity [15,85,86], inhibition of cell migration [87,90], induction of apoptosis [15,27,28,31,88], reversal of multidrug resistance [82,84], and favorable toxicity profiles [15,81,91,92]. Despite this progress, limited mechanistic investigation and the absence of clinical research continue to restrict the advancement of novel DHA-based hybrid therapies.

### 3.2. Drug Delivery Systems of DHA

DHA demonstrates broad antitumor efficacy, yet its clinical translation is challenged by low bioavailability, weak tumor targeting, formulation instability, and poor solubility. These issues collectively limit therapeutic potential. Development of efficient delivery platforms is therefore essential to improve performance and safety. Current research focuses on constructing diverse delivery systems based on liposomes, nanoparticles, and metal–organic frameworks. These platforms are increasingly integrated with multiple antitumor approaches, including chemodynamic therapy, photodynamic therapy, and immunotherapy, to overcome limitations of traditional formulations and achieve more precise and effective tumor treatment (Table 1).

#### 3.2.1. Drug Delivery Systems

Liposomes represent particularly promising carriers due to their excellent biocompatibility and stability. Ginsenoside Rg3-loaded liposomes significantly improve DHA release rates and stability while reducing systemic toxicity and enhancing antitumor activity [93]. Alkyl glycoside-modified liposomes increase tumor targeting and antitumor efficacy while maintaining favorable stability [94]. RGD-modified pH/ROS dual-responsive lipid nanoparticles provide controlled release of DHA [95]. DHA-TET liposomes, prepared by combining docosahexaenoic acid with tetracycline hydrochloride, enable targeted delivery, prolong circulation time, and improve therapeutic efficacy against breast cancer [96]. Although liposome technology is relatively mature (e.g., liposomal doxorubicin), next-generation functionalized liposomes tailored for DHA, including dual-responsive platforms and combination carriers, still require systematic preclinical safety evaluation and scalable manufacturing development.

Nano-delivery systems can achieve tumor-targeted accumulation through the EPR effect. CuO_2_@Cu-TA@DSF/DHA [99] and PTX-PEG-DHA nanoparticles [101] have demonstrated tumor-targeting activity in pancreatic cancer, cervical cancer, and colorectal adenocarcinoma. DHA-paclitaxel nano-delivery systems [116] and PEG-b-PLL-TK-DHA nanoparticles (OD-M) [115] release active agents in response to high ROS levels. Other nanoparticles, such as Ca/DHA@AFn [97] and BSA-AuNC-MnO_2_@DHA [100], improve DHA hydrophobicity, while FLD nanoparticles enhance blood–brain barrier penetration [98]. The literature also reports that modifying nano-delivery systems with DHA during intravenous administration may overcome major limitations—including poor targeting efficiency and rapid plasma clearance—thereby improving the in vivo delivery of nanomedicines [102]. For example, transferrin-micelle@SD undergoes uptake through transferrin receptor-mediated internalization and traffics to lysosomes. In the acidic lysosomal environment, transferrin undergoes a conformational change, releasing Fe^3+^, DHA, and sorafenib, generating ROS and accelerating ferroptosis [103]. However, heterogeneity in the tumor microenvironment—such as variable transferrin receptor expression and hypoxia gradients—may impair nanoparticle penetration and targeting accuracy. Future development should prioritize collaborative material optimization and engineering of more realistic biomimetic models to advance clinical translation.

Metal–organic frameworks have emerged as promising DHA carriers due to high drug-loading capacity, controlled release characteristics, and sensitivity to the tumor microenvironment. Among these, zeolitic imidazolate framework-8 systems show particularly strong performance: ZIF-8 loaded with DHA enhances targeting and antitumor activity in ovarian [104], liver [105], and lung cancer models [106]. Iron-doped ZIF-8 nanoparticles also demonstrate high DHA loading and significantly enhance therapeutic effects in liver cancer [107]. In addition, both in vitro and in vivo studies confirm that pHCT74/MOF-5@DHA&CORM-401 nanoparticles integrate the advantages of nanoparticle and MOF systems [108]. Despite these advances, biosafety concerns—including metal ion retention and long-term toxicity—and unclear in vivo metabolic profiles remain core barriers to clinical translation. Currently, only a limited number of MOF-based drugs have reached clinical trials.

Other delivery platforms have also shown potential. Exo-DHA complexes formed by loading DHA onto milk exosomes via ultrasonic processing enhance antitumor activity against triple-negative breast cancer and melanoma while improving oral absorption [112]. The ink@hydrogel-DHA system, constructed from traditional Chinese ink and agarose hydrogel, exhibits controlled drug release and strong anticancer activity [113].

Evidently, the development and application of drug delivery systems represent an effective approach to improving DHA bioavailability.

#### 3.2.2. DHA-Based Carrier Delivery System Therapy

Chemodynamic therapy generates highly toxic ROS through Fenton and Fenton-like reactions involving H_2_O_2_ within the tumor microenvironment. Studies show that the iron-based single-atom nanozyme Fe-SAE@D loaded with DHA provides high atomic utilization, well-defined active sites, and strong catalytic capacity [109]. The iron-derived MOF system DHA@MIL-101 enables high DHA loading and controlled release through its porous structure and large surface area [117]. The manganese-based nanosystem DHA@vhmMN@RM exhibits strong GSH responsiveness and biodegradability, allowing synchronized release of Mn^2+^ and DHA [118]. DSUC-Gel, which combines copper sulfide, DHA, and sulfasalazine, enhances chemodynamic therapy and induces ferroptosis [110]. However, this approach remains limited by insufficient endogenous H_2_O_2_ and high GSH levels.

PDT uses photosensitizers that, upon laser irradiation, react with oxygen to generate cytotoxic singlet oxygen (^1^O_2_), selectively destroying tumors. Co-encapsulating DHA and the photosensitizer dihydroporphyrin e6 within a PEG-PCL polymer enables responsive release in the acidic tumor microenvironment, enhancing targeting capability. This platform supports sequential sustained release of DHA and Ce6, initially rapid and then prolonged, preventing premature clearance. Under laser irradiation, it generates reactive oxygen species efficiently, thereby inducing tumor cell apoptosis [119]. The IR808/DHA-S-CA nanomicelle system employs a ROS-responsive prodrug design that enables precise drug release in the high-ROS environment characteristic of tumors. Leveraging IR808’s ability to generate ROS under near-infrared irradiation, it amplifies oxidative stress and enhances antitumor activity while reducing treatment toxicity [120].

Immunotherapy restores antitumor immunity through checkpoint inhibition, yet suppression within the tumor microenvironment and inadequate T-cell infiltration contribute to therapeutic resistance. Studies indicate that D@FMN-M induces ferroptosis in breast cancer cells and M2 macrophages, promoting polarization toward the M1 phenotype and reversing immune suppression [121]. ZnP@DHA/Pyro-Fe core–shell nanoparticles co-deliver Chol-DHA and Pyro-Fe, increasing CD8^+^ T-cell infiltration and significantly enhancing CRC sensitivity to anti-PD-L1 therapy [111]. pH/ROS dual-responsive PDBA@RSL-3 nanoparticles induce ferroptosis and activate T-cell immunity in a pancreatic ductal adenocarcinoma model, demonstrating synergy when combined with PD-L1 inhibitors [122]. These findings show that nanomedicine-based delivery systems provide novel strategies to overcome barriers in immunotherapy.

#### 3.2.3. Use with Other Preparations

Combining existing formulations with DHA enhances its antitumor activity. Studies show that zinc protoporphyrin-9 increases intracellular free heme, activates the peroxy bridge, and augments DHA-induced ROS production, thereby enhancing DHA sensitivity in melanoma B16 and breast cancer 4T1 cells [123]. Likewise, δ-aminolevulinic acid increases glioblastoma sensitivity to DHA by enhancing porphyrin production [124]. However, although elevated heme levels improve the antitumor activity of DHA, reports indicate that high heme levels in Plasmodium parasites impair the antimalarial activity of artemisinin derivatives [125]. This suggests that the therapeutic effects of artemisinin compounds exhibit a strong “context-dependent” nature, in which pharmacological outcomes reflect not only drug properties but also the metabolic state and defense mechanisms of target cells.

### 3.3. Combined Use as an Adjuvant Therapy

DHA can be combined with cancer treatments such as chemotherapy, radiotherapy, and immunotherapy, demonstrating substantial advantages (Table 2). Its core therapeutic value lies in synergistically enhancing anticancer efficacy and reducing toxicity through multiple mechanisms.

#### 3.3.1. Combination Use with Chemotherapy Drugs

##### Reversal of Tumor Cell Drug Resistance

Tumor cell drug resistance is a major obstacle to successful chemotherapy, severely compromising treatment efficacy and prognosis. DHA reverses drug resistance through multiple mechanisms. The literature reports that both DDA1 and p-STAT3 expression are significantly elevated in cisplatin-resistant cells. DHA simultaneously suppresses DDA1 expression, induces G0/G1 arrest, and inhibits p-STAT3 expression, thereby exerting anti-proliferative and pro-apoptotic effects [142]. In HER2-positive breast cancer, elevated phospho-TCTP correlates with poor response to trastuzumab. DHA restores trastuzumab sensitivity by reducing phospho-TCTP levels and blocking microtubule dynamics [135]. Additionally, elevated heme levels in osimertinib-resistant EGFR-mutant NSCLC impair heme metabolism and contribute to treatment resistance. DHA reverses this resistance by increasing ROS levels and downregulating HO-1 expression [143]. Reports also show that DHA induces marked apoptosis and ferroptosis in gefitinib-resistant A549 cells through ROS accumulation, thereby enhancing gefitinib sensitivity [144]. Accordingly, reversing drug resistance is a potential pathway for DHA to be involved in clinical tumor therapy.

##### Enhanced Chemosensitivity of Tumor Cells

DHA improves the cytotoxic response to chemotherapeutic drugs at doses that are otherwise minimally toxic to tumor cells, thereby increasing chemosensitivity. Studies report that DHA reduces clonogenic capacity and spheroid formation by inhibiting the AKT/mTOR pathway and reducing cancer stem-like properties, thereby increasing oxaliplatin sensitivity in CRC cells [145]. In lung cancer, co-treatment with cisplatin and DHA downregulates GPX4 and FTH1 while upregulating TFRC and ZIP14, resulting in more severe ferroptosis [132]. In vitro experiments revealed that DHA can also enhance the sensitivity of various tumor cells (such as MDA-MB-231 and U251) to ferroptosis induced by GPX4 inhibition by promoting HO-1-mediated mitochondrial oxidative stress and triggering a feedback loop of mitochondrial fusion [146]. In HCC, DHA disrupts the CCL2/TGF-β-mediated immunosuppressive microenvironment and reverses cisplatin-induced immunosuppression, thereby enhancing antitumor efficacy [128]. Additionally, low LASS2 expression correlates with poor bladder cancer prognosis, while DHA increases cisplatin sensitivity by upregulating LASS2 [147]. Notably, DHA can enhance tumor cell chemosensitivity through multiple pathways.

##### Synergistic Effects with Chemotherapeutic Agents

DHA exerts synergistic effects with chemotherapy through multiple mechanisms: ① ER stress pathway synergy. Studies show that DHA increases intracellular ROS by inhibiting PRDX2, activating ER stress, and synergizing with oxaliplatin to induce apoptosis in CRC cells [126]. DHA also increases CHOP expression, synergistically enhancing TRAIL-induced apoptosis in colon cancer cells through ER stress induction [127]. In NSCLC, DHA enhances ROS production by suppressing PTGS1, synergistically activating ER stress and MAPK pathways with cisplatin to exert antitumor activity [130]. DHA further promotes dendritic cell phagocytosis and CTL responses by activating the PERK/eIF2α pathway, increasing calreticulin exposure, and reversing poor immune activation when cisplatin is used alone [133]. ② Mitochondrial stress pathway synergy. In acute T-lymphoblastic leukemia, DHA inhibits Mcl-1 and Bcl-2 and synergizes with ABT-737 to activate mitochondrial apoptosis [148]. Similar synergy has been observed between DHA and genistein in acute myeloid leukemia [149]. In TNBC, DHA negatively regulates the STAT3/HIF-1α pathway, increases the Bax/Bcl-2 ratio, activates mitochondrial apoptosis, and enhances doxorubicin-induced apoptosis [137]. ③ Ferroptosis pathway synergy. In gastric cancer, DHA amplifies ROS while oridonin depletes intracellular GSH, jointly disrupting redox balance [115]. DHA also enhances cisplatin activity by inhibiting GPX4, promoting lipid peroxide accumulation, and ultimately inducing ferroptosis [140]. However, the antitumor effect of DHA combined with DDP may be histological subtype-dependent. In patient-derived NSCLC tissues, DHA combined with cisplatin significantly increases tumor cell death in lung squamous carcinoma but not lung adenocarcinoma, due to upregulated GPX4 expression in LUAD but not LUSC, making LUSC more sensitive to DHA-induced ferroptosis [134]. Notably, recent research has revealed that the combination of ascorbic acid and DHA successfully induces ferroptosis in LUAD cells (A549, H1299, LLC) by suppressing SLC7A11 expression and reducing GPX4 levels [150]. This contradiction suggests that the efficacy of the DHA combination strategy in LUAD may depend heavily on the choice of experimental model. The protective effect of the in situ tissue microenvironment may explain the resistance phenotype of LUAD in the patient-derived NSCLC tissues model, while cell line models detached from the microenvironment reveal its inherent ferroptosis sensitivity. Therefore, future studies should further validate the feasibility of ascorbic acid combined with DHA in models that more closely mimic physiological conditions. ④ Other synergistic mechanisms. Studies show that DHA degrades PHB2 via the ubiquitin pathway, blocking PHB2-mediated RCHY1 upregulation and p53/p21 downregulation, thereby synergizing with oxaliplatin against CRC [151]. DHA combined with resveratrol upregulates DLC1 and downregulates TCTP, suppressing Cdc42-mediated JNK/NF-κB signaling and synergistically inhibiting HCC migration [152]. DHA and anlotinib jointly downregulate Bcl-2 and VEGF-A expression, synergistically suppressing gastric cancer proliferation and migration [139]. DHA, in combination with the glutaminase inhibitor CB839, synergistically blocks glutamine metabolism, increases GBM apoptosis, and suppresses migration [153]. DHA-TF enhances DR5 expression when combined with DHER, synergistically inducing apoptosis in TNBC [154]. A disulfide-linked docetaxel-DHA co-delivery nanoplatform significantly promotes apoptosis in 4T1 cells, achieves anti-metastatic effects, and synergistically inhibits breast tumor growth while reducing toxicity to normal tissue [155]. Given this, there is substantial basic research evidence supporting the synergistic effects of DHA combined with chemotherapeutic agents, which raises the intriguing possibility that DHA may also have the potential to alleviate the side effects of chemotherapy.

##### Reduction in Drug Toxicity

DHA shows potential in mitigating the toxic side effects of chemotherapy drugs. Dose-dependent and cumulative nephrotoxicity are major limitations of cisplatin treatment. Studies indicate that DHA prevents cisplatin-induced nephrotoxicity by inhibiting NFκB p65-mediated inflammation and alleviating p63-mediated mitochondrial intrinsic and Fas receptor-associated extrinsic apoptosis pathways [156]. Doxorubicin is widely used for skin cancer therapy, but its severe cardiotoxicity restricts clinical application. Research indicates that the DHA–doxorubicin prodrug (DOX-S-DHA), synthesized through a single sulfur bond, may reduce cardiotoxicity while enhancing the synergistic antitumor effects of doxorubicin and DHA by bidirectionally regulating p53 [157]. These findings demonstrate that DHA can mitigate chemotherapy-related toxicity through multiple mechanisms and formulation approaches.

In summary, combining DHA with chemotherapy drugs holds strong potential for enhancing therapeutic benefit while reducing toxic side effects.

#### 3.3.2. Combined with Radiotherapy

DHA also demonstrates promise as a radiosensitizer. Reports show that in breast cancer, DHA enhances radiosensitivity by promoting ferroptosis through targeting the hsa_circ_0001610/miR-139-5p/SLC7A11 axis [158]. Simultaneously, DHA synergistically remodels the tumor immune microenvironment during radiotherapy. In NSCLC, DHA downregulates PD-L1 by inhibiting TGF-β, p-AKT, and p-STAT3 signaling, thereby blocking immune escape. It also activates TRIM21 and modulates EMT-associated proteins, synergistically enhancing radiotherapy response [129]. Additionally, radiation induces RNF126 upregulation in TNBC, promoting DNA repair and radioresistance. DHA disrupts the HER2-AKT-NF-κB pathway, inhibits RNF126 expression, and improves radiotherapy efficacy [159]. In the A549 radiation-resistant model, DHA suppresses PINK1/Parkin-mediated mitophagy by downregulating CIRBP, thereby reducing radiation tolerance [131]. DHA also activates Nrf2/HO-1 signaling, increases GPX4 and GSH levels, inhibits ferroptosis, and protects against radiation-induced lung injury [160]. However, the radiosensitizing effect of DHA may be dependent on tumor type or treatment regimen. A study in a CRC model using CT26 cells demonstrated that DHA combined with low-fractionation radiotherapy (6 Gy × 3 fractions) did not further enhance the antitumor efficacy of radiotherapy. There was no significant difference in tumor volume or weight between the combination therapy group and the radiotherapy-alone group [161]. It is evident that the efficacy of combining DHA with radiotherapy remains controversial, and further exploration of its applicable conditions is required.

#### 3.3.3. Combination with Immunotherapy

Immunotherapy harnesses the immune system to eradicate tumors, with immune checkpoint inhibitors such as anti-PD-1 antibodies serving as core treatment options for solid tumors by restoring the function of exhausted immune cells. DHA enhances tumor sensitivity to anti-PD-1 therapy through multiple mechanisms. Studies suggest that abnormal tumor vasculature contributes to the development of an immunosuppressive microenvironment, undermining the efficacy of immunotherapy. In breast cancer models, DHA alleviates tumor-associated vascular abnormalities through the TNF-α pathway, improving anti-PD-1 treatment responses [136]. Additionally, abnormally high expression of YAP1 in HCC correlates with resistance to anti-PD-1 therapy. YAP1 promotes lipid droplet accumulation within tumor cells, accelerates disease progression, and suppresses immune responses by inducing exhaustion of peripheral CD4^+^/CD8^+^ T cells. DHA suppresses YAP1 expression, reduces lipid droplet accumulation, and improves the tumor metabolic environment [162]. DHA also enhances anti-PD-1 efficacy by downregulating PD-L1 and increasing intratumoral T-cell infiltration [163]. Furthermore, DHA increases the abundance of Bacteroides fragilis by inhibiting YAP1, restoring immune balance, and improving HCC responsiveness to anti-PD-1 therapy [164]. However, the upstream signaling pathways and specific targets through which DHA regulates YAP1 remain to be defined.

## 4. Preliminary Studies of Clinical Anticancer Effects

### 4.1. Preclinical Trials

#### 4.1.1. Pharmacokinetic Study of DHA

DHA is the active metabolite of artemisinin and is characterized by rapid absorption and elimination. Studies report the following: ① After oral administration of its prodrug artemether, DHA is rapidly absorbed and converted into its active form, with peak plasma concentrations typically occurring within 2 hours; intravenous administration shortens the time to peak levels to within 25 minutes. ② DHA shows broad tissue distribution with an apparent volume of distribution of approximately 0.5–1.0 L/kg [165]. A study conducted under near-physiological conditions showed that DHA binding to serum albumin is driven by a negative enthalpy change and a positive entropy change, with hydrophobic interactions being the main contributing force [166]. ③ In humans, DHA is mainly metabolized through conjugation reactions mediated by the UDP-glucuronosyltransferase system, with UGT1A9 and UGT2B7 identified as the major enzymes [167]. In mice, DHA also undergoes hydroxylation and OH dehydration, in addition to glucuronidation [168]. Notably, DHA exerts mixed inhibitory effects on several major cytochrome P450 enzymes, including CYP1A2, CYP2B6, CYP2C9, CYP2C19, CYP2D6, and CYP3A4. This inhibition may increase plasma concentrations of drugs metabolized through these enzymes, thereby elevating the risk of adverse drug reactions [169]. ④ DHA metabolites are cleared from plasma and excreted by the kidneys. They display a relatively high clearance rate (0.5–1.5 L/kg/h) and a short elimination half-life of approximately 0.5–1.5 h, resulting in limited systemic retention. These pharmacokinetic features may be attributed to DHA’s low polarity and poor water solubility associated with its peroxy bridge and lactone ring structure [165]. Collectively, these factors restrict DHA’s bioavailability, targeting efficiency, and stability, thereby limiting its therapeutic potential.

#### 4.1.2. Safety Studies on DHA

As research expands, potential risks associated with DHA—including hepatotoxicity, reproductive toxicity, and neurotoxicity—have gained attention. In hepatotoxicity studies, a 28-day gavage experiment in SD rats showed no toxicity in either male or female rats at 25 mg/kg DHA, while a 50 mg/kg dose was safe only for males. A high dose of 60 mg/kg induced significant hepatotoxicity in both sexes, evidenced by elevated ALT, increased liver weight, and mild vacuolation [170]. In reproductive toxicity studies, DHA inhibited polar body extrusion in porcine oocytes. DHA concentrations of 10 μM and 20 μM slightly reduced Pb1 extrusion, while 40 μM and 80 μM caused a sharp decline [171]. Whole-embryo culture studies in rats showed that exposure to 0.01–2 μg/mL DHA during the early or late stages of a 48-hour culture period disrupted yolk sac hematopoiesis and significantly reduced erythrocyte numbers [172]. Likewise, 2 μM DHA exposure in erythrocyte precursor cells inhibited growth and delayed differentiation [173]. In neurotoxicity studies, adult Swiss white mice administered 300 mg/kg/day DHA for 28 days showed impairment in brainstem auditory and vestibular reflex pathways. No significant neurotoxicity was observed in animals treated with doses below 200 mg/kg/day [174]. These findings suggest that DHA-associated risks may depend on multiple variables, including drug concentration, exposure duration, and species differences. Therefore, more research is required to determine optimal dosing strategies to minimize toxicity and support safe clinical application.

### 4.2. Clinical Trials

The major goal of translational research is to advance safe and effective therapies into clinical use. DHA currently has established standard dosing in antimalarial regimens. In the dihydroartemisinin-piperaquine regimen, the recommended dose for adults and children weighing ≥ 25 kg is 4 mg/kg dihydroartemisinin once daily (with 18 mg/kg piperaquine) for 3 consecutive days. However, clinical evidence in oncology remains extremely limited. Using the keywords “Dihydroartemisinin and Tumor,” a ClinicalTrials.gov search identified eight clinical studies (as of 1 February 2026) (Table 3). Among these, three have been completed: two list polycystic ovary syndrome as the primary indication, with “tumor” mentioned only in the background, and the third focuses on artesunate pessary, with DHA appearing only in the background. Thus, clinical trials explicitly testing DHA for tumor indications remain in early stages.

Given the limited clinical trials on DHA’s antitumor effects, we also reviewed studies on artemether. Research indicates that artemether can be used to treat solid tumors such as NSCLC [175] and CRC [176], with good tolerability. Pharmacokinetic analysis of oral artemisinin shows that its active metabolite, DHA, undergoes stable metabolism, although apparent clearance increases over time [177]. Regarding safety, clinical studies have reported potential hematologic toxicity associated with artemisinin derivatives. For example, some breast cancer patients experienced leukopenia, neutropenia, and anemia after taking the drug orally [178]. Some solid tumor patients who received intravenous artemisinin developed febrile neutropenia, allergic reactions, and abnormal liver function [179]. These findings suggest that artemether has demonstrated antitumor potential in clinical trials, implying that DHA may hold significant value in cancer treatment. However, due to the adverse reactions observed with artemether—including ototoxicity and hematologic toxicity—future DHA trials must prioritize monitoring these risks. In summary, antitumor research on DHA remains in its early stages, and current evidence remains insufficient. Future work should include in-depth chronic toxicology studies, drug interaction evaluations, and large-scale clinical trials to fully assess DHA’s therapeutic value.

**Table 3:** Clinical Trials Identified with the Keywords “Dihydroartemisinin and Tumor” at ClinicalTrials.gov.

NO	Study Title	Conditions	Status	Identifier	Ref.
1	Pharmacokinetics of Intravaginal, Self-administered Artesunate Vaginal Pessaries Among Women in Kenya	Cervix Cancer	Completed, Phase 1	NCT06263582	[180]
2	Efficacy of Dihydroartemisinin for Treating PCOS	Polycystic Ovary Syndrome	Completed, Phase 2	NCT06417099	[181]
3	The Effect of Dihydroartemisinin in PCOS	Polycystic Ovary Syndrome	Completed, Phase 4	NCT05465135	[182]
4	Phase II Study of Artesunate Ointment for the Treatment of Vulvar High Grade Squamous Intraepithelial Lesions (Vulvar HSIL, VIN2/3)	Vulvar Diseases, HPV Infection	Recruiting, Phase 2	NCT06075264	[183]
5	Dihydroartemisinin for the Treatment of Polycystic Ovary Syndrome	Polycystic Ovary Syndrome	Recruiting, Phase 2	NCT06842524	[184]
6	Artesunate Ointment for the Treatment of Anal HSIL in HIV-negative Participants	Anal High-grade Squamous Intraepithelial Lesion	Recruiting, Phase 2	NCT06206564	[185]
7	Safety and Effectiveness Study of Pre-operative Artesunate in Stage II/III Colorectal Cancer (NeoART-V)	Colorectal Cancer	Unknown, Phase 2	NCT03093129	[186]
8	Artesunate Suppositories for the Treatment of HIV-negative Patients with Intra-anal HSIL	Anal High Grade Squamous Intraepithelial Lesion	Not recruiting, Phase 2	NCT05555862	[187]

## 5. Summary and Prospects

The persistent increase in global tumor incidence and mortality presents a serious threat to human health. Current treatment modalities continue to face significant challenges, including high recurrence rates, limited curative efficacy, and substantial adverse effects. Consequently, developing novel antitumor agents that are safe, effective, and well-tolerated has become both a worldwide research priority and an urgent clinical need.

DHA, the highly active metabolite of artemisinin derived from *Artemisia annua*, has attracted considerable attention due to its broad biological activity and favorable safety profile. This review summarizes the diverse pharmacological mechanisms of DHA demonstrated in preclinical research, including inhibition of proliferation, induction of programmed cell death, suppression of metastasis, inhibition of angiogenesis, reprogramming of tumor energy metabolism, and modulation of the tumor immune microenvironment. It also outlines emerging strategies to enhance DHA’s antitumor potential and examines its use as an adjunctive therapy, underscoring its potential as a promising anticancer agent. When combined with chemotherapy, radiotherapy, and immunotherapy, DHA offers advantages, including reversal of tumor drug resistance, enhanced treatment sensitivity, synergistic effects, and mitigation of treatment-related toxicity. Additionally, approaches such as drug delivery system incorporation and molecular hybridization with other pharmacophores may improve DHA’s bioavailability and pharmacokinetic profile.

Despite its confirmed antitumor efficacy, several challenges impede the clinical translation of DHA. Modern pharmacological studies demonstrate that while DHA exhibits broad antitumor activity in phenotypic screening, its precise molecular targets and mechanisms remain incompletely elucidated. Employing advanced methodologies, including activity-based protein profiling, network pharmacology, bioinformatics, and multi-omics technologies, represents a promising strategy to rapidly identify its molecular targets and delineate associated signaling networks. Future investigations should also address the stereospecific activity of DHA enantiomers. Additional research directions should utilize organoid models to examine tumor resistance mechanisms, explore DHA’s effects on multicellular interactions within the tumor immune microenvironment, and evaluate its therapeutic response across diverse cancer types to advance precision oncology. The effects of DHA on tumor stem cells and glycolytic metabolism also merit further investigation. Importantly, as a small molecule with multifaceted pharmacological activities, DHA should be explored for novel applications in complex tumor-associated comorbidities, accompanied by mechanistic studies to understand these therapeutic effects.

Second, DHA’s poor aqueous solubility, susceptibility to oxidative degradation, short half-life, and low bioavailability necessitate advanced delivery technologies, structural modifications, or rationally designed combination therapies to maximize therapeutic efficacy while minimizing treatment-related adverse effects. However, the metabolic profile of DHA in vivo remains inadequately characterized, and its oral metabolism demonstrates substantial interspecies variation, highlighting the need for more human-relevant model systems. Furthermore, while drug delivery systems improve DHA’s bioavailability and pharmacokinetics, their component materials or structural modifications may alter its mechanism of action compared to unformulated DHA. Therefore, further investigation into the mechanisms underlying both approaches is essential. The endoperoxide bridge constitutes DHA’s core cytotoxic pharmacophore, which remains relatively inert until activated by ferrous iron (Fe^2+^). Consequently, integrating DHA with transferrin-mediated delivery systems has emerged as a highly promising strategy to enhance tumor-specific accumulation while reducing off-target effects. Although large-scale DHA production currently depends on semi-synthetic processes involving artemisinin extraction from *Artemisia annua* followed by chemical reduction, biosynthetic approaches utilizing engineered yeast present a viable alternative worthy of continued development.

Notably, DHA not only suppresses established tumor growth but may also exert effects in early carcinogenesis. Studies indicate that in Helicobacter pylori-associated gastric cancer models, DHA blocks early carcinogenic events by inhibiting pathogen colonization, reducing ROS-mediated oxidative damage in gastric epithelial cells, and suppressing inflammatory signaling pathways such as NF-κB, ultimately lowering gastric cancer incidence [188]. In a colitis-associated colorectal cancer model, DHA prevents early carcinogenesis by reducing macrophage infiltration and inflammatory cytokines (TNF-α, IL-6, and IFN-β) through inhibition of the TLR4 pathway in macrophages [189]. Furthermore, DHA promotes Treg proliferation, suppresses Th1 and Th17 differentiation, and regulates the Th17/Treg balance, thereby attenuating intestinal inflammation and preventing colon cancer initiation and progression [77]. These findings suggest that DHA may regulate early carcinogenic processes. However, its effects on canonical carcinogenic pathways—such as DNA damage protection, mutation prevention, or genomic stability—remain unclear. Future research should investigate DHA’s actions during tumor initiation to determine its potential role in cancer chemoprevention.

Ultimately, the clinical value of DHA must be validated through rigorously designed clinical trials that address its pharmacokinetic characteristics, optimal indication selection, resistance mechanisms, and combination therapy optimization. Concurrently, careful attention should be directed toward evaluating DHA’s chronic toxicity profile and potential drug interactions. Through interdisciplinary collaboration, DHA demonstrates considerable potential to become an important component of comprehensive cancer treatment, providing patients with safer and more effective therapeutic options.

In summary, extensive preclinical evidence and emerging clinical trial data provide substantial support for DHA’s application in oncology. These findings also reinforce and advance clinical research on DHA within modern pharmaceutical formulations.

## Figures and Tables

**Table 1:** Application of dihydroartemisinin DDS in the treatment of tumors.

DDS	Animal/Cell Model		Advanced Effect	Ref.
ginsenoside Rg_3-based liposomes loaded with DHA and paclitaxel	In vitro	MDA-MB-231, 4T1, and H9c2 cells	Better stability. High release rate. Low side effect. Stronger anti-tumor effect.	[93]
alkyl glycoside-modified dihydroartemisinin liposomes	In vitro	HepG2 cells	Better stability. Targeting ability. Stronger anti-tumor effect.	[94]
RLNP/DC	In vitro In vivo	HCT116 and SW480 cells BALB/c nude mice	Better stability. Targeting ability. Stronger anti-tumor effect.	[95]
DHA-TET pH-sensitive LPs	In vitro	MCF-7 cells	Stronger anti-tumor effect. High encapsulation efficiency.	[96]
Ca/DHA@AFn	In vitro	4T1 cells	Increasing drug loading efficiency. Targeting ability. Stronger anti-tumor effect.	[97]
FLD NPs	In vitro	U251 cells	Stronger anti-tumor effect. Crossing the blood–brain barrier easily.	[98]
CuO_2_@Cu-TA@DSF/DHA	In vitro	PANC-1 and BxPC-3 cells	Targeting ability. Stronger anti-tumor effect.	[99]
BSA-AuNC-MnO_2_@DHA	In vitro	AML-12 and HK-2 cells	Better stability. Good biocompatibility	[100]
PD@PPD NPs	In vitro in vivo	HT-29 cancer cells Balb/c nude mice	Good biocompatibility.	[101]
PPD NPs	In vitro In vivo	4T1 and 3T3 cells Tumor-harboring mice	Efficient chemotherapy and minimum off-target toxicities	[102]
Tf-Mic@SD	In vitro	A549 cells	Stronger anti-tumor effect. Targeting ability.	[103]
ZIF-DHA	In vitro In vivo	SKOV3 and A2780 cells Female BALB/c nude mice	Tumor-inhibiting activity.	[104]
DHA@ZIF-8	In vitro	HepG2	Better stability. Good biocompatibility. Low side effect. Stronger anti-tumor effect.	[105]
D-ZIF	In vitro In vivo	A549-TAX cells BALB/c nude mice	Low side effect. Targeting ability. Stronger anti-tumor effect.	[106]
CDZs	In vitro In vivo	Hep G2, HCT116, MCF-7, RAW264.7, and U937 cells BALB/c nude mice	Increasing drug loading efficiency. Targeting ability. Stronger anti-tumor effect.	[107]
MOF-5@DHA&CORM-401 NPs	In vitro in vivo	CT26 cells BALB/c mouse	Stronger anti-tumor effect in the combination with ICD.	[108]
Fe-SAE@D	In vitro	GL261	Lower costs. Better stability.	[109]
CuS NPs	In vitro in vivo	4T1 cells, L929 cells BALB/c mice	Stronger anti-tumor effect in the combination with ICD. Excellent biosafety.	[110]
ZnP@DHA/Pyro-Fe	In vitro In vivo	MC38 and CT26 cells SD rats, BALB/c and C57BL/6 mice	Low side effect. Stronger anti-tumor effect. Targeting ability.	[111]
Exo-DHA	In vitro	MDA-MB-23 and 4T1 cells	Easy to release. Stronger anti-tumor effect.	[112]
ink@hydrogel and DHA	In vitro In vivo	4T1 cells BALB/c nude mice	Controllable drug release. Stronger anti-tumor effect.	[113]
Exo-DHA	In vitro	B16F10 cells	Stronger anti-tumor effect. Improving oral bioavailability.	[114]
NPs OD-M	In vitro	AGS cells	Good biocompatibility. Stronger anti-tumor effect.	[115]

**Table 2:** Application of dihydroartemisinin combined with other therapies in the treatment of tumors.

Disease	Animal/Cell Model	Types	Routes	Dose	Effects and Related Mechanism	Ref.
Colon cancer	HCT116 cells RKO cells athymic BALB/c nu/nu male mice	In vitro In vitro In vivo	- - ip	DHA: 5 µM Oxaliplatin: 60 µM DHA: 15 µM Oxaliplatin: 60 µM DHA: 5 mg/kg Oxaliplatin: 2 mg/kg	↑ ROS, ATF4, p-eIF2α, p-JNK ↓ PRDX2, p-STAT3	[126]
	HCT116 cells	In vitro	-	DHA: 50 µM TRAIL: 2 ng/mL	↑ CHOP, Cleaved PARP, and ATF4	[127]
Liver cancer	DEN/TCPOBOP-induced liver tumor model in male C57BL/6 mice	In vivo	ip	DHA: 25 mg/kg DDP: 2 mg/kg	↓ TGF-β, CCL2	[128]
Lung cancer	A549, PC9, and Lewis lung cancer cells (LLC) A549/X and PC9/X cells female-specific pathogen-free (SPF) C57/BL6 mice	In vitro In vivo	- ip	Radiate: 2, 4, and 6 Gy DHA: 50 mg/kg Radiate: 2 Gy	↓ PD-L1, TGF-β, PI3K/AKT, and STAT3 signaling pathways ↑ Trim21 and EMT-Related Proteins	[129]
	A549 cells H460 cells H460 subcutaneous xenograft BALB/c nude mice	In vitro In vitro In vivo	- - ip	DHA: 20 µM DDP: 30 µM DHA: 20 µM DDP: 30 µM DHA: 5 mg/kg DDP: 4 mg/kg	↑ ROS, p-JNK, p-eIF2α, ATF4, p-p38 ↓ PTGS1	[130]
	radioresistant lung cancer A549 cells	In vitro	-	Hydroxychloroquine: 50 µM DHA: 8 µM Radiate: 2 Gy	↓ PD-L1, AKT/GSK3β/cyclinD1 Pathway, CIRBP ↑ LC 3 II, ROS	[131]
	A549/H1975 cells A549/H1975 subcutaneous xenograft C57 BL/6 mice	In vitro In vivo	- ip	DHA: 20 µM DDP: 10 µM DDP: 10 mg/kg DHA: 20 mg/kg	↑ ZIP 14, TFRC ↓ GPX 4, FTH 1	[132]
	LLC BALB/c mice	In vitro In vivo	- ip	CDDP: 100/150 µM DHA: 10 µM	↑ ROS, HMGB1, IFN-γ	[133]
	LUSC cells derived from NSCLC patients	In vitro	-	DDP: 3 µM DHA: 10 µM	↑ Bax/Bcl-2	[134]
Breast cancer	HCC1954, HCC1569, BT-474 cells CB17SCID mice	In vitro In vivo	- ip	DHA: 2.5 µM T-DM1: 0.25 µg/mL T-DM1: 10 mg/kg DHA: 25 mg/kg	↓ AKT phosphorylation levels, TCTP ↑ p-AMPK	[135]
	4T-1 subcutaneous xenograft of BALB/c nude mice	In vivo	ip ig	Anti-PD-1: 100 µg/mouse DHA: 50 mg/kg	↓ TNF-α, CD31, VEGFA, CD34	[136]
	MD-AMB-231 cells	In vitro	-	DHA: 50 µM Dox: 0.5 µmol/L	↑ Cleaved Caspase 3, Cleaved PARP, PCNA, Bax/Bcl-2 ↓ p-STAT3, p-JAK1/2, Bcl-XL, Mcl-1, HIF-1α	[137]
	4T-1 cells 4T-1 subcutaneous xenograft of BALB/c nude mice	In vitro In vivo	- ip	ZnPPIX: 10 µM DHA: 2 µM DHA: 50 mg/kg ZnPPIX: 25 mg/kg	↑ ROS	[123]
Leukemia	U937 cells KG-1 cells	In vitro In vitro	- -	DHA: 14.95 µM ABT: 0.12 µM DHA: 11.26 µM ABT: 0.18 µM	↑ Bax, Cyt C, Cleaved Caspase 9, Cleaved Caspase 3 ↓ Bcl-2	[138]
Gastric cancer	SGC7901 gastric cancer cells	In vitro	-	Anlotinib: 2.5 µmol/L DHA: 5 µmol/L	↓ Ki67, Bcl-2, and VEGF-A	[139]
	AGS tumor-bearing mice	In vivo	iv	DHA: 15 mg/kg ORI: 15 mg/kg	↑ ROS ↓ GSH	[115]
	GC cells	In vitro	-	DDP: 1.5–15 µM DHA: 3–100 µM	↓ GPX4, GSH, GSH-PX ↑ROS, MDA	[140]
Endometrial carcinoma	Ishikawa cells	In vitro	-	DHA: 40 µM DPP: 20 µM	↑ Cleaved Caspase-3	[141]
Glioblastoma	human neuroblastoma cells (SHSY5Y)	In vitro	-	DHA: 0.5 µM 5-ALA: 0.25 mM	↑ ROS	[124]

↓ indicates inhibition/reduction, while ↑ indicates increase/promotion; ip, Intraperitoneal; ig, Intragastric; iv, Intravenous injection.

## Data Availability

No new data were created or analyzed in this study. Data sharing is not applicable to this article.

## Supplementary Materials

The following supporting information can be downloaded at https://www.mdpi.com/article/10.3390/ijms27083420/s1.

## Abbreviations

The following abbreviations are used in this manuscript:

AKT	Protein kinase B
AMPK	AMP-dependent protein kinase
Bad	B-cell lymphoma-2-associated death-inducing factor
Bax	Bcl-2-associated X protein
Bcl-2	B-cell lymphoma-2
CCL2	C-C motif chemokine ligand 2
CDK	Cyclin-dependent kinase
CDT	Chemodynamic therapy
cGAS	Cyclic guanosine monophosphate-adenosine monophosphate synthase
CHOP	C/EBP homologous protein
CIZ1	CDKN1A-binding zinc finger protein 1
CRC	Colorectal cancer
Caspase	Cysteinyl aspartate-specific proteinase
DDP	Cisplatin
DDS	Drug delivery systems
DHA	Dihydroartemisinin
DOX	Doxorubicin
eIF2α	Eukaryotic initiation factor 2α
EMT	Epithelial–mesenchymal transition
ER	Endoplasmic reticulum
ERK1/2	Extracellular regulated protein kinases
FGF2	Fibroblast growth factor 2
GPX4	Glutathione peroxidase 4
GSDME	Gasdermin E
GSH	Glutathione
HCC	Hepatocellular carcinoma
HER2	Human epidermal growth factor receptor 2
HIF-1α	Hypoxia-inducible factor-1α
HO-1	Heme Oxygenase-1
ICD	Immunogenic cell death
ICIs	Immune checkpoint inhibitors
IFN-β	Interferon-β
IL-6	Interleukin-6
JNK	c-Jun N-terminal kinase
LASS2	Late endosomal/lysosomal adaptor, MAPK, and mTOR activator 2
LPO	Lipid peroxides
MAPK	Mitogen-activated protein kinase
Mcl-1	Myeloid cell leukemia-1
MMP-9	Matrix metalloproteinase-9
MMPs	Matrix metalloproteinases
MOFs	Metal–organic frameworks
mTOR	Mammalian target of rapamycin
NDDSs	Nanodelivery systems
NSCLC	Non-small cell lung cancer
ORI	Oridonin
OSCC	Oral squamous cell carcinoma
PARP	Poly-ADP-ribose polymerase
PCa	Prostate cancer
PCD	Programmed cell death
PDT	Photodynamic therapy
PERK	Protein kinase R-like endoplasmic reticulum kinase
PI3K	Phosphoinositide 3-kinase
Rb	Retinoblastoma protein
ROS	Reactive Oxygen Species
ROR1	Receptor tyrosine kinase-like orphan receptor 1
STAT3	Signal transducer and activator of transcription 3
Smad	Mothers against decapentaplegic homolog
TCTP	Translation control tumor protein
TGF-β	Transforming growth factor-β
TLR4	Toll-like receptor 4
TME	Tumor microenvironment
TNBC	Triple-negative breast cancer
TNF-α	Tumor necrosis factor-α
TRAIL	Tumor necrosis factor-related apoptosis-inducing ligand
TfR	Transferrin receptor
VEGF	Vascular endothelial growth factor
VM	Vascular mimicry
YAP1	Yes-associated protein 1
ZIF-8	Zeolite imidazole framework-8
