# Supplementary material for: The Therapeutic Potential of Dihydroartemisinin in Cancer Treatment
Source: Int J Mol Sci. 2026 Apr 10;27(8):3420. doi: 10.3390/ijms27083420 (PMC13116139; doi:10.3390/ijms27083420)
Supplement: Supplementary file 1 [file ijms-27-03420-s001.zip › ijms-4212689-supplementary.pdf]

Supplementary Table

Table S1: Anticancer hybrids of dihydroartemisinin.

No.	Chemical formula	Model	$IC_{50}/GI_{50}/ED_{50}/LC_{50}$			Compared with the parent compound	Ref.
				DHA	Derivative		
1	（**1, Figure 9**）	A549 cells	IC_{50}	69.4 μM	8.32 μM	Enhanced cytotoxic activity against lung cell lines.	[81]
2	（**2, Figure 9**）	A549 cells	IC_{50}	69.4 μM	21.6 μM	Enhanced cytotoxic activity against lung cell lines.	[81]
3	（**3, Figure 9**）	A549 cells	IC_{50}	69.4 μM	7.54 μM	Enhanced cytotoxic activity against lung cell lines.	[81]
4	（**4, Figure 9**）	A549 cells	IC_{50}	69.4 μM	16.3 μM	Enhanced cytotoxic activity against lung cell lines.	[81]
5	（**5, Figure 9**）	A549 cells	IC_{50}	69.4 μM	12.0 μM	Enhanced cytotoxic activity against lung cell lines.	[81]
6	（**6, Figure 9**）	A549 cells	IC_{50}	69.4 μM	22.7 μM	Enhanced cytotoxic activity against lung cell lines.	[81]
7	（**7, Figure 9**）	A549 cells	IC_{50}	69.4 μM	25.6 μM	Enhanced cytotoxic activity against lung cell lines.	[81]
8	（**8, Figure 9**）	A549 cells	IC_{50}	69.4 μM	16.3 μM	Enhanced cytotoxic activity against lung cell lines.	[81]
9	（**9, Figure 9**）	A549 cells	IC_{50}	69.4 μM	44.7 μM	Enhanced cytotoxic activity against lung cell lines.	[81]
10	（**10, Figure 9**）	A549 cells	IC_{50}	69.4 μM	9.10 μM	Enhanced cytotoxic activity against lung cell lines.	[81]
11	（**11, Figure 9**）	A549 cells	IC_{50}	69.4 μM	15.8 μM	Enhanced cytotoxic activity against lung cell lines.	[81]
12	（**12, Figure 9**）	MCF-7, MDA-MB-231, MCF-7/ADR and MDA-MB-231/ADR cells	IC_{50}	73.2,69.6, 79.1,82.8 μM	19.4, 15.3, 20.1,18.8 μM	Profound activity against both drug-sensitive and drug-resistant breast cancer cell lines.	[82]
13	（**13, Figure 9**）	MCF-7, MDA-MB-231, MCF-7/ADR and MDA-MB-231/ADR cells	IC_{50}	73.2, 69.6, 79.1,82.8 μM	27.2, 19.7, 33.3,21.6 μM	Profound activity against both drug-sensitive and drug-resistant breast cancer cell lines.	[82]
14	（**14, Figure 9**）	MCF-7, MDA-MB-231, MCF-7/ADR and MDA-MB-231/ADR cells	IC_{50}	73.2, 69.6, 79.1,82.8 μM	39.1, 43.3, 38.4,42.9 μM	Profound activity against breast cancer cell lines.	[83]
15	（**15, Figure 9**）	MCF-7, MDA-MB-231, MCF-7/ADR and MDA-MB-231/ADR cells	IC_{50}	73.2, 69.6, 79.1,82.8 μM	57.0, 49.6 47.7,69.2 μM	Profound activity against breast cancer cell lines.	[83]
16	（**16, Figure 9**）	MCF-7, MDA-MB-231, MCF-7/ADR and MDA-MB-231/ADR cells	IC_{50}	73.2, 69.6, 79.1,82.8 μM	30.8, 26.0, 42.3,27.4 μM	Profound activity against breast cancer cell lines.	[83]

17	(**17, Figure 9**)	MCF-7, MDA-MB-231, MCF-7/ADR MDA-MB-231/ADR cells	IC_{50}	73.2, 69.6, 79.1,82.8 μM	18.9, 25.3, 20.7,26.6 μM	Profound activity against breast cancer cell lines.	[83]
18	(**18, Figure 9**)	MCF-7, MDA-MB-231, MCF-7/ADR MDA-MB-231/ADR cells	IC_{50}	73.2, 69.6, 79.1,82.8 μM	52.2,38.1, 35.8,44.3 μM	Profound activity against breast cancer cell lines.	[83]
19	(**19, Figure 9**)	MCF-7, MDA-MB-231, MCF-7/ADR MDA-MB-231/ADR cells	IC_{50}	73.2, 69.6, 79.1,82.8 μM	57.6, 50.2, 49.9,58.0 μM	Profound activity against breast cancer cell lines.	[83]
20	(**20, Figure 9**)	MCF-7, MDA-MB-231, MCF-7/ADR and MDA-MB-231/ADR cells	IC_{50}	73.2, 69.6, 79.1,82.8 μM	26.6, 35.7, 30.8,29.5 μM	Profound activity against breast cancer cell lines.	[83]
21	(**21, Figure 10**)	MCF-7, MDA-MB-231, MCF-7/ADR and MDA-MB-231/ADR cells	IC_{50}	73.2, 69.6, 79.1,82.8 μM	19.8, 24.4, 27.9,20.1 μM	Profound activity against breast cancer cell lines.	[83]
22	(**22, Figure 10**)	MCF-7, MDA-MB-231, MCF-7/ADR and MDA-MB-231/ADR cells	IC_{50}	73.2, 69.6, 79.1,82.8 μM	43.2, 29.6, 36.2,28.8 μM	Profound activity against breast cancer cell lines.	[83]
23	(**23, Figure 10**)	MCF-7, MDA-MB-231, MCF-7/ADR and MDA-MB-231/ADR cells	IC_{50}	73.2, 69.6, 79.1,82.8 μM	23.7, 30.9, 33.3,27.4 μM	Profound activity against breast cancer cell lines.	[83]
24	(**24, Figure 10**)	MCF-7, MDA-MB-231, MCF-7/ADR and MDA-MB-231/ADR cells	IC_{50}	73.2, 69.6, 79.1,82.8 μM	15.1, 18.3 16.2,17.9 μM	Profound activity against breast cancer cell lines.	[83]
25	(**25, Figure 10**)	MCF-7, MDA-MB-231, MCF-7/ADR, and MDA-MB-231/ADR cells	IC_{50}	73.2, 69.6, 79.1,82.8 μM	33.5, 30.9, 28.0,45.7 μM	Profound activity against breast cancer cell lines.	[83]
26	(**26, Figure 10**)	MCF-7, MDA-MB-231, MCF-7/ADR, and MDA-MB-231/ADR cell	IC_{50}	73.21, 69.60, 79.10, 82.78 μM (mean)	49.51, 51.16, 22.97, 48.97 μM (mean)	Profound activity against triple-negative breast cancer and overcome the drug resistance.	[84]
27	(**27, Figure 10**)	MCF-7, MDA-MB-231, MCF-7/ADR, and MDA-MB-231/ADR cells	IC_{50}	73.21, 69.60, 79.10, 82.78 μM (mean)	3.90, 3.84, 10.18, 2.85 μM (mean)	Profound activity against triple-negative breast cancer and overcome the drug resistance.	[84]

28	（**28, Figure 10**）	A549, Bel-7402, HCT-116, SW620 and LO2 cells	IC_{50}	19.38, 43.16, 5.10, 6.61, 8.43 μM (mean)	1.00, 8.41, 0.31, 0.60, 16.78 μM (mean)	Enhanced cytotoxic activity against lung and colorectal cancer lines and was non-toxic to normal human liver cells LO2.	[15]
29	（**29, Figure 10**）	HT-29, HCT-116 and FHC cells	IC_{50}	-	10.95, 11.85, >25 μM (mean)	Enhanced cytotoxic activity against colorectal cancer lines and was non-toxic to non-tumorigenic (FHC) colonocytes.	[31]
30	（**30, Figure 10**）	MCF-7, A549, HepG-2, MDA-MB-231 and L02 cells	IC_{50}	34.29, 25.04, 31.71, 58.69, 40.21 μM (mean)	7.47, 5.07, 6.96, 7.88, 31.73 μM (mean)	Enhanced cytotoxic activity against breast and liver cancer lines and was non-toxic to normal human liver cells LO2.	[27]
31	（**31, Figure 10**）	MCF-7 and MDA-MB-231cells	IC_{50}	28.28,48.80,14.85,82.78 μM	13.63,14.87 μM	Enhanced cytotoxic activity against breast cell lines.	[91]
32	（**32, Figure 10**）	MCF-7 and MDA-MB-231 cells	IC_{50}	28.28,48.80,14.85,82.78 μM	18.09,15.13 μM	Enhanced cytotoxic activity against breast cell lines.	[91]
33	（**33, Figure 10**）	MCF-7 and MDA-MB-231 cells	IC_{50}	28.28,48.80,14.85,82.78 μM	1.27,15.06μM	Enhanced cytotoxic activity against breast cell lines.	[91]
34	（**34, Figure 10**）	MCF-7, MDA-MB-231, MCF-7/ADR and MDA-MB-231/ADR cells	IC_{50}	73.21,69.60,79.10,82.78 μM	7.79,8.13,6.26,9.15 μM	Potential to overcome drug-resistant breast cancer cell lines. Excellent safety and selectivity profiles.	[92]
35	（**35, Figure 10**）	MCF-7, MDA-MB-231, MCF-7/ADR and MDA-MB-231/ADR cells	IC_{50}	73.21,69.60,79.10,82.78 μM	3.85,5.19,9.36,7.25 μM	Potential to overcome drug-resistant breast cancer cell lines. Excellent safety and selectivity profiles.	[92]
36	（**36, Figure 10**）	MCF-7, MDA-MB-231, MCF-7/ADR and MDA-MB-231/ADR cells	IC_{50}	73.21,69.60,79.10,82.78 μM	9.53,8.14,3.21,4.99 μM	Potential to overcome drug-resistant breast cancer cell lines. Excellent safety and selectivity profiles.	[92]
37	（**37, Figure 10**）	BGC-823, HCT-116 and MCF-7 cells	IC_{50}	29.64,41.69,34.32 μM (mean)	12.48,13.60,18.24μM (mean)	Decreased the protein levels of apoptosis-related proteins in gastric cancer BGC-823 cells and liver cancer HepG-2 cells.	[86]
38	（**38, Figure 10**）	BGC-823, HCT-116 and MCF-7 cells	IC_{50}	29.64,41.69,34.32 μM (mean)	16.31,21.68,33.67μM (mean)	Decreased the protein levels of apoptosis-related proteins in gastric cancer BGC-823 cells and liver cancer HepG-2 cells.	[86]
39	（**39, Figure 10**）	BGC-823, HCT-116 and MCF-7 cells	IC_{50}	29.64,41.69,34.32 μM (mean)	8.30,16.83,24.27 μM (mean)	Decreased the protein levels of apoptosis-related proteins in gastric cancer BGC-823 cells and liver cancer HepG-2 cells.	[86]
40	（**40, Figure 10**）	T24 cells	IC50	71.5 μM (mean)	3.2 μM (mean)	Enhanced cytotoxic activity against bladder cancer cell lines.	[28]

41	（**41, Figure 10**）	HepG2 and Huh-7 cells	IC50	22.7 μM (mean)	1.00 μM (mean)	Enhanced cytotoxic activity against liver cancer cell lines.	[88]
42	（**42, Figure 10**）	4T1, CT26, HeLa, and MCF-7 cells	IC50	4.5,4.44,5.66.24 μM	1.3,1.78,2.59,1.32μ M	Enhanced ability to inhibit breast cancer metastasis and growth.	[45]
43	（**43, Figure 10**）	HCT116 and RKO cells	IC50	10.355,5.538 μM (mean)	0.692,1.478 μM (mean)	Enhanced ability to inhibit colorectal cancer metastasis and growth. Enhanced cytotoxic activity against colorectal cancer cell lines.	[90]
44	（**44, Figure 10**）	HCT116 and RKO cells	IC50	10.355,5.538 μM (mean)	0.523,1.539 μM (mean)	Enhanced ability to inhibit colorectal cancer metastasis and growth. Enhanced cytotoxic activity against colorectal cancer cell lines.	[90]
45	（**45, Figure 10**）	HCT116 and RKO cells	IC50	10.355,5.538 μM (mean)	0.727,0.856 μM (mean)	Enhanced ability to inhibit colorectal cancer metastasis and growth. Enhanced cytotoxic activity against colorectal cancer cell lines.	[90]
46	（**46, Figure 10**）	HCT116 and RKO cells	IC50	10.355,5.538 μM (mean)	0.819,1.056 μM (mean)	Enhanced ability to inhibit colorectal cancer metastasis and growth. Enhanced cytotoxic activity against colorectal cancer cell lines.	[90]
